# Anti-Influenza Activity of the Ribonuclease Binase: Cellular Targets Detected by Quantitative Proteomics

**DOI:** 10.3390/ijms21218294

**Published:** 2020-11-05

**Authors:** Vera Ulyanova, Raihan Shah Mahmud, Alexander Laikov, Elena Dudkina, Maria Markelova, Ahmed Mostafa, Stephan Pleschka, Olga Ilinskaya

**Affiliations:** 1 Institute of Fundamental Medicine and Biology, Kazan Federal University, 420008 Kazan, Russia; ulyanova.vera@gmail.com (V.U.); alexander.laikov@yandex.ru (A.L.); lenatimonina@rambler.ru (E.D.); mimarkelova@gmail.com (M.M.); olga.ilinskaya@kpfu.ru (O.I.); 2 Institute of Medical Virology, Justus Liebig University Giessen, 35392 Giessen, Germany; ahmed_elsayed@daad-alumni.de (A.M.); stephan.pleschka@viro.med.uni-giessen.de (S.P.); 3 Center of Scientific Excellence for Influenza Viruses, National Research Centre, Cairo 12622, Egypt

**Keywords:** influenza virus, ribonuclease, binase, antiviral activity, proteomics, liquid chromatography-tandem mass spectrometry (LC-MS/MS), multiple reaction monitoring (MRM)

## Abstract

Unpredictable influenza pandemics, annual epidemics, and sporadic poultry-to-human avian influenza virus infections with high morbidity and mortality rates dictate a need to develop new antiviral approaches. Targeting cellular pathways and processes is a promising antiviral strategy shown to be effective regardless of viral subtypes or viral evolution of drug-resistant variants. Proteomics-based searches provide a tool to reveal the druggable stages of the virus life cycle and to understand the putative antiviral mode of action of the drug(s). Ribonucleases (RNases) of different origins not only demonstrate antiviral effects that are mediated by the direct RNase action on viral and cellular RNAs but can also exert their impact by signal transduction modulation. To our knowledge, studies of the RNase-affected cell proteome have not yet been performed. To reveal cellular targets and explain the mechanisms underlying the antiviral effect employed by the small extra-cellular ribonuclease of *Bacillus pumilus* (binase) both in vitro and in vivo, qualitative shotgun and quantitative targeted proteomic analyses of the influenza A virus (IAV) H1N1pdm09-infected A549 cells upon binase treatment were performed. We compared proteomes of mock-treated, binase-treated, virus-infected, and virus-infected binase-treated cells to determine the proteins affected by IAV and/or binase. In general, IAV demonstrated a downregulating strategy towards cellular proteins, while binase had an upregulating effect. With the help of bioinformatics approaches, coregulated cellular protein sets were defined and assigned to their biological function; a possible interconnection with the progression of viral infection was conferred. Most of the proteins downregulated by IAV (e.g., AKR1B1, AKR1C1, CCL5, PFN1, RAN, S100A4, etc.) belong to the processes of cellular metabolism, response to stimulus, biological regulation, and cellular localization. Upregulated proteins upon the binase treatment (e.g., AKR1B10, CAP1, HNRNPA2B1, PFN1, PPIA, YWHAB, etc.) are united by the processes of biological regulation, cellular localization, and immune and metabolic processes. The antiviral activity of binase against IAV was expressed by the inversion of virus-induced proteomic changes, resulting in the inhibition of virus-associated processes, including nuclear ribonucleoprotein export (NCL, NPM1, Nup205, and Bax proteins involved) and cytoskeleton remodeling (RDX, PFN1, and TUBB) induced by IAV at the middle stage of single-cycle infection in A549 cells. Modulation of the immune response could be involved as well. Overall, it seems possible that binase exerts its antiviral effects in multiple ways.

## 1. Introduction

Influenza A virus (IAV) is an enveloped, single-stranded, negative-sense RNA virus. Its genome consists of eight RNA segments encoding 10 to 20 different known proteins required for viral replication and pathogenesis [1]. The accumulation of point mutations (antigenic drift) due to the error-prone viral polymerase is responsible for the continuous evolution of IAV, while segment recombination during coinfections (antigenic shift) accounts for the emergence of potentially pandemic viruses [2]. These genomic changes even allow the virus to overcome species barriers. In 2009, a novel IAV strain (H1N1pdm09) emerged in Mexico as a result of a reassortment among swine, avian, and human IAVs. H1N1pdm09 rapidly spread across the world and caused the first pandemic of the 21st century. It was characterized by efficient transmissibility, low virulence, and an inability to induce strong innate immune response in humans [3]. Now, H1N1pdm09 is established in human populations and circulates as a seasonal IAV. Seasonal IAV annually affect millions of people worldwide, causing mild to severe illness. The World Health Organization estimates that 290,000 to 650,000 people die each year from influenza virus-related respiratory diseases alone. The ability of H1N1pdm09 to reassort with cocirculating IAV strains and to accumulate mutations could potentially lead to the emergence of a highly pathogenic variant [2,4]. This demonstrates that IAV represent a global problem, despite the limited number of anti-influenza virus strategies applied.

For the prevention of IAV infections, vaccination is essential and can reduce illness and lessen the severity of infection. However, it is only effective when the vaccine is well-matched to the circulating strains [5,6]. Antigenic drift and interspecies transmission complicate the choice of the optimal strains for vaccine production [7]. This can be further complicated by unpredictable changes of the vaccine strain during production [6]. Currently, several classes of drugs are either licened or approved for anti-influenza treatment and prophylaxis in certain countries [1]. Adamantanes (amantadine and rimantadine; FDA-approved) target the viral M2 protein required for virus uncoating during replication. Neuraminidase inhibitors (e.g., oseltamivir and zanamivir, FDA-approved) impair the release of the virus from infected host cells. Indole derivatives (e.g., Umifenovir, approved in Russia and China) inhibit the IAV hemagglutinin-mediated fusion of the viral and endosome membranes. RNA-dependent RNA polymerase inhibitors (e.g., Baloxavir and Favipiravir, licensed in Japan, FDA-approved) suppress viral replication. As mentioned, IAV is able to develop drug resistance and escape selection pressure [1,8]. Nowadays, circulating IAV are resistant to M2 ion channel inhibitors (adamantanes), and their resistance to neurominidase inhibitor oseltamivir has already been reported [8,9]. Novel approaches in combating IAV are therefore urgently needed. Flavonoids and naphthoquinones from natural sources have been intensively studied as alternative antiviral drugs [10,11]. Microencapsulation technology for the delivery of antiviral small interfering RNA (siRNA) was also shown to be an efficient tool for silencing IAV infection [12]. In addition to broad-spectrum neutralizing anti-hemagglutinin monoclonal antibodies, inhibitors of the viral polymerase complex and compounds targeting host cell factors to alter the ability of the virus to replicate efficiently are currently under clinical trials [13,14,15]. Drugs aimed at cellular targets can effectively inhibit different IAV strains and minimize the emergence of resistant species [16]. Enzymes like ribonuclease from microbial origin were previously shown to interact with cellular signaling proteins and alter cellular biological processes demonstrating antiviral or antitumor activities [17,18]. These enzymes could be a promising tool to specifically target intracellular processes in infected cells.

Many ribonucleases are known for their antiviral properties. Four out of eight human canonical RNases possess antiviral activities, namely RNase-1 (RNase A), RNase-2 (eosinophil-derived neurotoxin), RNase-3 (eosinophil cationic protein), and RNase 5 (Angiogenin) [19]. RNase-3 nucleases from diverse kingdoms represent an ancient mechanism for the antiviral defense against positive-, but not negative-, stranded viruses [20]. Antiviral activity of the RNase-L was demonstrated against many viruses—in particular, RNA viruses; however, IAV is hardly affected, since its NS1 protein sequesters double-stranded (ds)RNA, thereby preventing the activation of RNase-L [21]. Representatives of the N1/T1/U2 family of microbial ribonucleases also demonstrate antiviral effects towards human viruses. At the same time, they have an important advantage over human RNases: they are not susceptible to the actions of the cytosolic ribonuclease-1 inhibitor, which protects RNA from invading ribonucleases [22]. Binase, a member of the N1/T1/U2 ribonuclease family, is an extracellular RNase of *Bacillus pumilus*. Only a minority of *Bacillus* species produce homologous RNases. Under natural conditions, binase is induced upon phosphate starvation in *Bacillus* [23]. Originally, the function of binase was the recovery of carbon, nitrogen, and phosphorous from alternative sources and the scavenging of ribonucleosides [24]. Recently, it was found that binase participates in the counteraction to bacteriophage infection [25]. Antiviral activity of binase towards eukaryotic viruses was already noticed as early as the 1980s [26]. Binase suppressed viral infections in animal models and decreased the lethality [26,27,28]. Recently, we studied the antiviral effects of binase towards reovirus [28,29], herpes virus [30], coronavirus [31], rhinovirus [32], and influenza virus [30,32,33,34] in cell cultures. The antiviral effects of binase were found to be the most profound against RNA viruses. In cell cultures, binase showed antiviral effects at concentrations as low as 10 µg/mL and exhibited cytotoxic properties only at concentrations higher than 400 µg/mL. Binase was found to degrade IAV RNAs within the infected cells, with no apparent effect on the cellular mRNAs of house-keeping genes [34]. Nevertheless, whole-transcriptome or proteome studies of binase-treated cells to assess the possible effects of binase on the host proteome have not been performed so far. Moreover, to our knowledge, the proteomic changes induced in cells by other RNases have also not been explored yet.

Here, we studied changes in the proteome profile of H1N1pdm09 virus-infected human adenocarcinoma alveolar basal epithelial (A549) cells upon binase treatment at the middle stage of a viral single-cycle infection (6 h postinfection). To determine the molecular features of the antiviral binase activity, we compared proteomes of mock-treated, binase-treated, virus-infected, and virus-infected binase-treated cells, then grouped the expressed proteins according to the effects of the virus and/or binase (upregulation, downregulation, or no change), and finally, analyzed those groups that reflected opposite activities caused either by the virus or binase. This is the first report of a whole proteomic analysis of the IAV-infected cell response following binase treatment in a cultured human cell model.

## 2. Results

### 2.1. Confirmation of Viral Infection of A549 Cells and Antiviral Action of Binase

**Figure 1:**
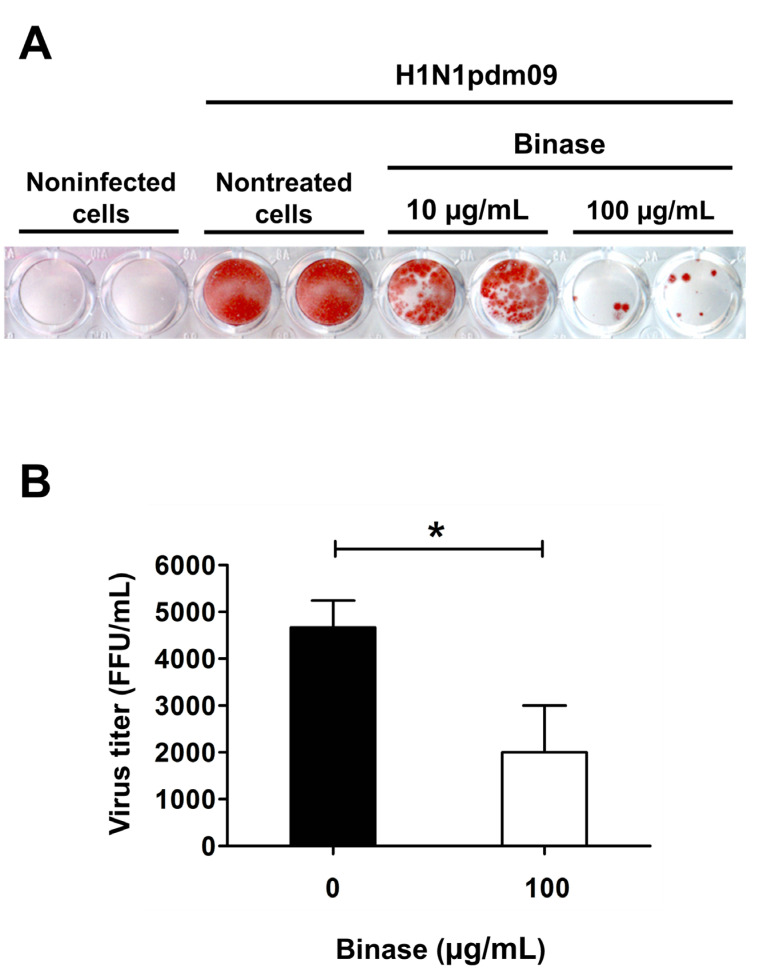
Detection of the influenza virus in the infected A549 cells. (**A**) The virus titer in the supernatant of H1N1pdm09-infected A549 cells (multiplicity of infection (MOI) = 1) treated with or without binase (10^4^ and 10^5^ U/mL) for 8 h postinfection (p.i.) was determined by a focus assay (*n* = 3 × 2). Infected MDCK-II cells are stained red. (**B**) The antiviral effect of binase was quantified, showing that the binase treatment reduced the virus titer by 57% (* *p* < 0.05).

To study proteomic changes induced by binase in IAV-infected cells, we chose human lung adenocarcinoma epithelial (A549) cells, which are commonly used as a human in vitro model for IAV infection and replication. The successful infection of A549 cells was verified by titration of the infectious particles using the focus assay, which is based on the detection of the IAV nucleoprotein (NP) in MDCK cells (Figure 1). The results indicate a profound antiviral effect at 100 μg/mL binase. Previously, binase was shown to demonstrate an antiviral effect against IAV during single- (12 h) and multiple (24 h)-cycle replications in A549 cells [33]. Additionally, it was demonstrated that binase penetrates into A549 cells within one h, and after three h, it is detected in the nuclei. At 4–6 h post-treatment (p.t.), it induces a temporal increase in the cellular permeability of A549 cells and apoptosis at 24 h p.t. [35]. As A549 cells infected by H1N1pdm09 undergo apoptosis at 24 h postinfection (p.i.) as well [36], resulting in concurrent apoptotic effects caused by the virus infection and by binase treatment, the discovery of antiviral binase-specific targets could be complicated. Therefore, samples for proteome profiling were taken at 6 h p.i. The chosen time point allowed registering the changes that took place during a single replication cycle of the virus (6–8 h) and corresponded to the stage when viral ribonucleoprotein complexes (RNPs) started to translocate from the nucleus to the cell membrane for virus assembly and the budding of progeny viral particles. To assure almost 100% cell infection upon proteome profiling, a multiplicity of infection (MOI) = 3 was used.

### 2.2. Qualitative Shotgun Proteomic Profiling of Virus-Infected and Binase-Treated Cells

**Figure 2:**
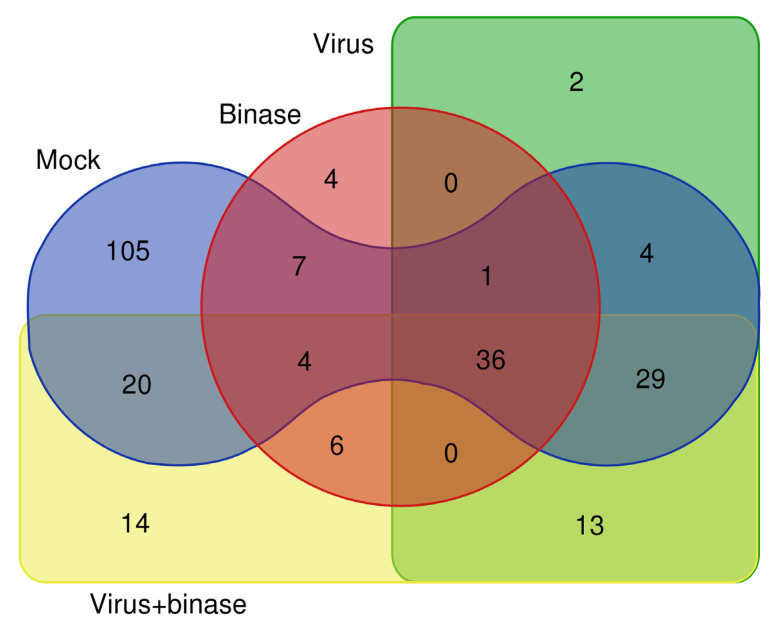
Venn diagram demonstrating the distribution of proteins identified by shotgun liquid chromatography-tandem mass spectrometry (LC-MS/MS) between the total cell fractions of A549 cells upon virus infection and binase treatment (mock-treated, binase-treated, virus-treated, virus and binase-treated, and preincubation scheme of the cell treatment).

To understand the molecular basis underlying binase activity against IAV in the infected A549 cells, we analyzed proteomic profiles of the cells treated in four different ways: (i) mock-infected cells (M), (ii) binase-treated cells (B), (iii) H1N1pdm09 virus-infected cells (V), and (iv) cells infected by the H1N1pdm09 and treated with binase (VB). Initially, a qualitative shotgun proteomics approach using liquid chromatography-tandem mass spectrometry (LC-MS/MS) was applied for the global characterization of the proteomic changes in the virus-infected and binase-treated cells, as well as for the selection of proteins for a further targeted quantitative mass spectrometry-based proteomics assay. For the qualitative shotgun proteomics analysis, the preincubation treatment scheme (PI), which included the step of virus incubation with binase prior to cell infection, was used (for details, see Section 2.3). We expected that such treatment would result in more pronounced proteomic changes, since it was shown previously that the preincubation of IAV with binase before infection significantly reduces the viral titer after single-cycle replication of the virus [32,33]. As it was shown that binase does not affect the virion [34], the enhanced antiviral effect is probably due to the fact that binase is consequently also present during infection. A shotgun proteomic analysis revealed that the majority of the identified cellular proteins were affected either by the virus or binase (Figure 2).

Proteins specifically suppressed by H1N1pdm09 at 6 h p.i. are related to the biological process of cellular component organization and include VIM, PFN1, TBB8L, TUBB3, TUBB4A, POTEKP, CLTCL1, HSPA8, and AKR1C1 (Table S1). Three proteins are involved in the negative regulation of supramolecular fiber organization (PFN1, HSPA8, and TUBB4A). Primary metabolic processes (oxidoreductases AKR1C1 and LDHA and isomerase PGAM1) are targeted as well. Protein synthesis that are activated by the virus are predominantly enriched in the process of cellular protein localization (TOMM5, GOLPH3L, AKT1, FAF2, EHD2, GNPAT, IPO4, MAPRE1, and MYO1C). Proteins involved in protein biosynthesis (ATAD3A, CMSS1, and EIF3L); DNA double-strand break repair (PRKDC); the inhibitor of protein Ser/Thr phosphatase 1 (PPP1R14B); and aldehyde dehydrogenase ALDH16A1 are also upregulated.

Proteins specifically suppressed by binase at 6 h p.t. are enriched primarily in a process of nucleobase-containing compound metabolism (GO:0006139) and the response to organic substance stimulus (GO:0071310). They belong to ribose phosphate and nucleotide metabolisms (PGK1, ENO1, GPI, TALDO1, TKT, and NDUFS8); mRNA metabolism (SF3B1, EFTUD2, HNRNPA2B1, RPS2, and RPL15); translation (EEF1A1, RPL15, RPS2, TARS, and TARSL2); and the epigenetic regulation of gene expression (ACTB and SF3B1). Proteins upregulated by binase have less in common and are represented by such proteins as kinesin KIF5B, heat shock protein DNAJB1, ATP-dependent RNA helicase DDX18, syntaxin-binding protein STXBP2, and paraoxonase PON2 (Table S1).

To characterize the modulation of cellular proteins by binase treatment during IAV infection, we compared V- and VB group samples (Table S1). In virus-infected cells, binase was found to downregulate proteins involved in the biological process of localization and the response to stimulus (HSPA2, TUBB2A, MAPRE1, and PPP1R14B), as well as the activator of 90-kDa heat shock protein (AHSA1), 60S ribosomal protein L15 (RPL15), and thioredoxin TXNDC5. Upregulated proteins were more numerous and belonged to different cellular processes, of which the most represented were involved in cellular component organization and localization (ACTC1, AP1B1, TUBB, TUBA1A, RDX, and MSN). The IAV-opposing activity of binase involves the upregulation of aldo-keto reductase AKR1C1, clathrin heavy-chain CLTCL1, profilin PFN1, and beta-actin-like protein POTEKP. Binase, when added to mock-treated or IAV-infected cells, brought about the elevation of paraoxonase PON2, clathrin interactor 1 CLINT1, kinesin KIF5B, methionine-tRNA ligase MARS, glycogen phosphorylase PYGB, and the translation initiation factor EIF3A.

### 2.3. Quantitative Proteomic Profiling of Virus-Infected and Binase-Treated Cells

A multiple-reaction monitoring (MRM) as a powerful high-resolution and high-precision-targeted MS technique was chosen for the accurate quantitation of proteins in the cell lysates. The results of LC-MS/MS were used as a guide for selecting protein targets for analysis by liquid chromatography-tandem mass spectrometry with multiple-reaction monitoring mode (LC-MRM/MS). Proteins not only notably varied between the samples, as revealed by shotgun proteomics, but, also, other proteins of potential interest, according to the literature, and those detected in the samples below the threshold were included in the analysis. A total number of 200 proteins were chosen. Out of these, 75 proteins were excluded at the verification step due to low signal intensity or as they were lacking unique peptides allowing their unambiguous detection in a complex mixture.

**Figure 3:**
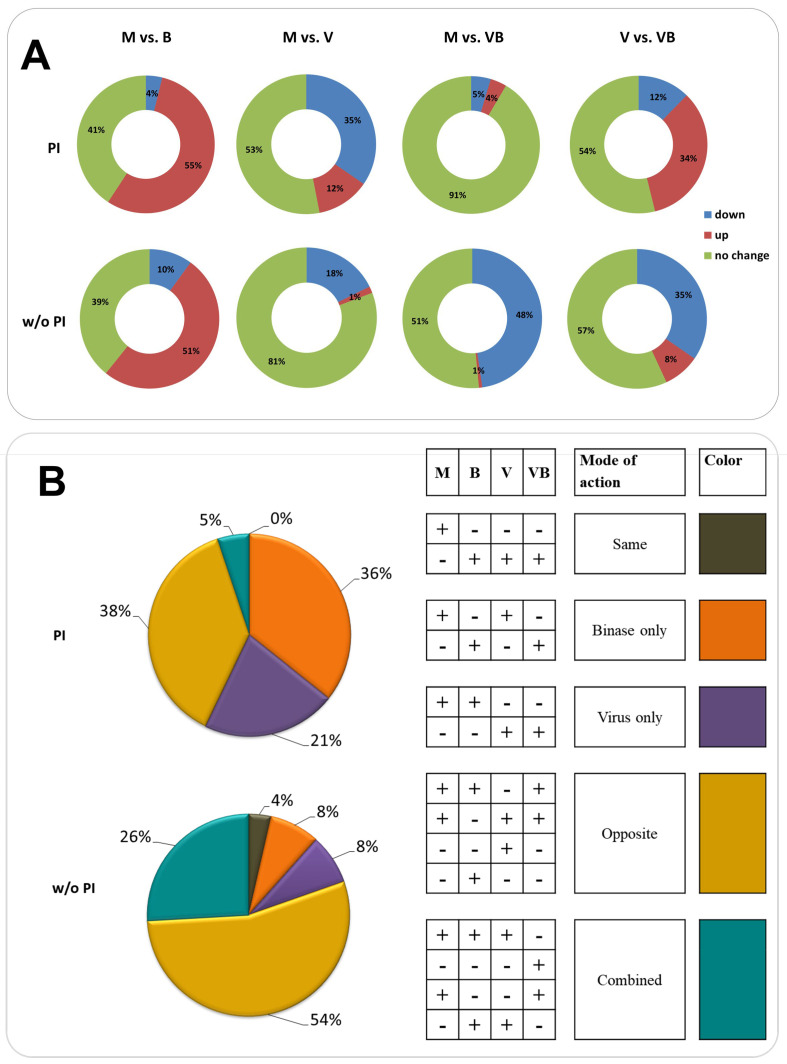
Distribution of proteins between cell samples upon virus infection and binase treatment as determined by targeted LC-MS. (**A**) Distribution of up- and downregulated proteins in cells upon virus infection and binase treatment. M: mock-treated, B: binase-treated, V: virus-treated, and VB: virus and binase-treated). PI: binase and virus were preincubated prior to cell administration and without (w/o) PI: binase was applied to cells after their infection with the virus. (**B**) Distribution of proteins identified in total cell fractions of A549 cells upon virus infection and binase treatment and differing between the samples in their quantity according to the mode of virus and binase actions. The mode of action was assumed on the basis of differences in the quantitative contents of certain proteins between the M, B, V, and VB sample groups. The plus sign indicates that the protein was present or increased (*p*-Val ≤ 0.05) in the sample, while the minus sign indicates that the protein was absent or decreased (*p*-Val ≤ 0.05). The coloring for the chart is related to the colors of groups in the right table, which were formed based on the mode of action of the virus and/or binase. Same action means that the same proteins were changed by the influenza A virus (IAV) infection or binase treatment (grey). When the proteins were significantly changed only in the samples treated by binase, they were grouped into the category “binase only” (orange). The same rule was applied for “virus only” proteins (purple). Opposite action means that effect of IAV on the cell proteome was flipped over by the presence of binase and vice versa (yellow). The combined action of IAV and binase was assessed when changes were induced by the simultaneous presence of IAV and binase in the cell and could not be addressed exactly to the IAV or binase (turquoise).

For a targeted proteomic analysis, two schemes of cell treatments differing by the mode of binase application were used. The PI scheme included a step of virus preincubation with binase before cell infection. In brief, virus, binase, and their mixture were preincubated in phosphate-buffered saline (PBS) for 30 min and then applied to the A549 cells. After one h, cells were washed and grown in the fresh media (M and V) or binase-containing media (B and VB) for 6 h. A without (w/o) PI scheme implied the binase treatment of cells after IAV infection. In this case, binase was present in the media only during 6 h of cell growth. Virus preincubation with binase (PI) greatly changed the proteome profiles of virus-infected cells (Figure 3A). In general, binase increased the protein levels in A549 cells, while IAV had an opposite effect. Despite the fact that more cellular proteins were affected by IAV under the PI scheme as compared to the w/o PI scheme (Figure 3A, M vs. V), 90% of the studied proteins in the VB-treated cells were the same as in the mock (Figure 3A, M vs. VB), suggesting the considerable impact of binase.

The virus or binase targeted different proteins in A549 cells (Figure 3B). Most of the proteins downregulated by IAV belong to the processes of cellular metabolism, response to stimulus, biological regulation, and cellular localization (Table S2). IAV reduced the level of metabolic proteins, such as cofactor and small-molecule metabolic proteins, as well as proteins enriched in the biological process of immune response (Table 1). Proteins involved in cytoskeleton organization, transport, and localization were decreased as well (Table 1). Upregulated proteins belong to leucocyte activation and protein-containing complex assembly (Table 1). An activating effect of IAV was registered mainly in the PI scheme. Without preincubation (w/o PI), only two proteins were significantly increased: ANXA2 and RPL15, which are related to virus replication and translation.

Binase decreased the amount of proteins not related to a distinct biological process but combined by a cellular component, the membrane (LRPPRC, TRAP1, HSPD1, PHB2, SURF4, NDUFS8, KRT10, PON2, MYOF, and RAP1A)—predominantly, a mitochondrial membrane—as well as CNP and YLPM1 proteins. The effect was obvious in the w/o PI scheme (Table S2). After cell preincubation with binase, binase reduced the amount of CS, PHB2, SURF4, and YLPM1 proteins. Numerous upregulated proteins are united by the processes of biological regulation, including the regulation of gene expression, exocytosis, endocytosis, cellular localization, immune process and oxidation reduction, and metabolic processes (Table 1). Many of the proteins belong to the group mediating the cellular response to organic substances and stimuli.

**Figure 4:**
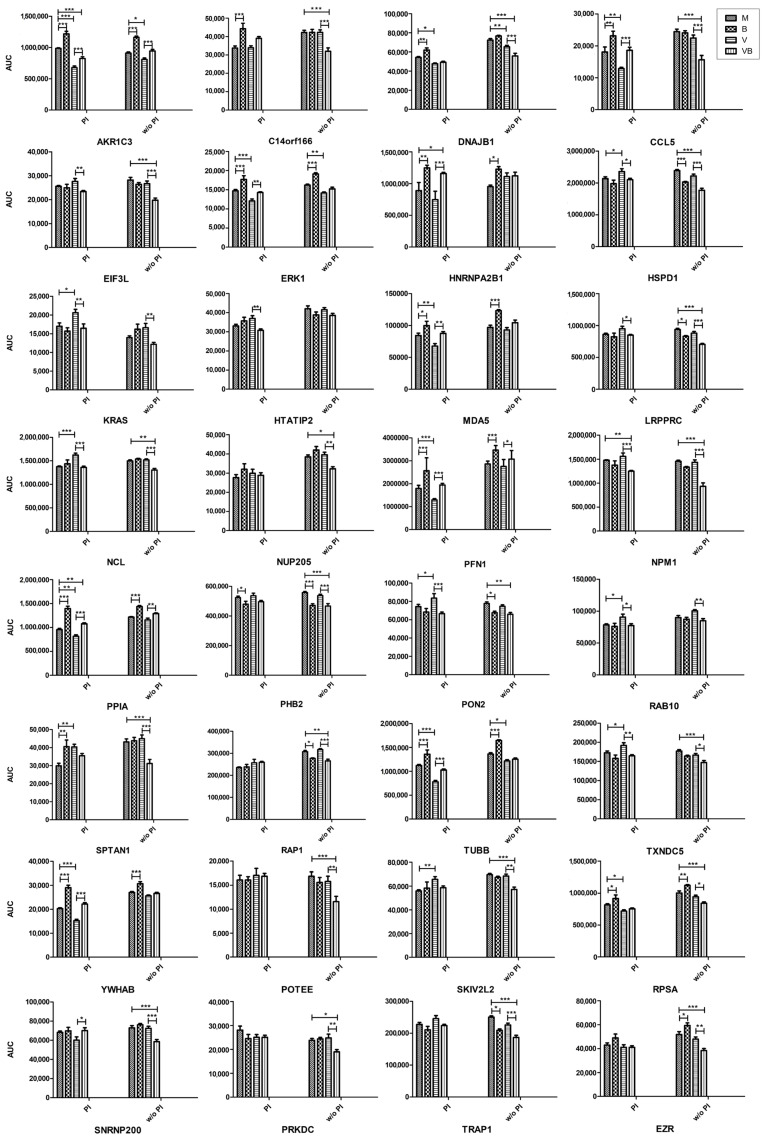
Relative protein quantities in mock-treated (M), binase-treated (B), virus-infected (V), and virus-infected binase-treated (VB) samples as determined by targeted liquid chromatography-tandem mass spectrometry with multiple-reaction monitoring mode (LC-MRM/MS). PI: binase and virus were preincubated prior to cell administration and w/o PI: binase was applied to cells after their infection with the virus. AUC: area under the curve. * *p* < 0.05, ** *p* < 0.01, *** *p* < 0.001.

It is worth mentioning that, independently of the treatment scheme, binase decreases the level of PHB2 and YLPM1 proteins and activates numerous similar proteins; all of them are different from those affected by the virus. IAV demonstrates a downregulating strategy towards cellular proteins. Despite the specific cell treatment scheme, IAV decreases the amount of such proteins as TOMM5, SORL1, S100A4, AKR1C3, DNAJB1, TUBB4B, TUBB, PGAM1, LDHA, MAPK1, and MAPK3 (Figure 4). Interestingly, these proteins, except for TOMM5, are always upregulated by binase. This IAV-opposing tendency is seen both under PI and w/o PI schemes, where 38 out of 42 and 12 out of 23 IAV-decreased proteins, respectively, are upregulated by binase. The amount of only two proteins, ANXA2 and RPL15, under the w/o PI scheme and 16 proteins under the PI scheme is increased by IAV. Some of them are also increased by binase (ANXA2, RPL15, and SPTAN1), but no IAV-opposing activity was found.

To specifically address the changes induced by binase on the proteome of the IAV-infected cell that presumably mediate the antiviral effect of binase, we compared the V and VB samples. Under the PI scheme, the biggest changes were attributed to the PFN1, YWHAB, S100A4, HNRNPA2B1, and POTEI proteins, which were increased in the VB sample, as compared to the V sample, by more that 1.5-fold, and to the ATL3, EIF3A, EIF3L, KRAS, NCL, PON2, NPM1, ANXA2, and HTATIP2 proteins, which were decreased by 0.7–0.8-fold (Table S2 and Figure 4). Under the w/o PI scheme, the AKR1B1, AKR1B10, AKR1C1, AKR1C3, PGK1, and TOMM5 proteins were 1.2–1.3-fold increased, while the CCL5, CLINT1, EIF3L, KRAS, NPM1, POTEE, RPL15, SPTAN1, MAPRE1, and SURF4 proteins were 0.6–0.7-fold decreased by binase in VB vs. V cells (Table S2 and Figure 4).

**Figure 5:**
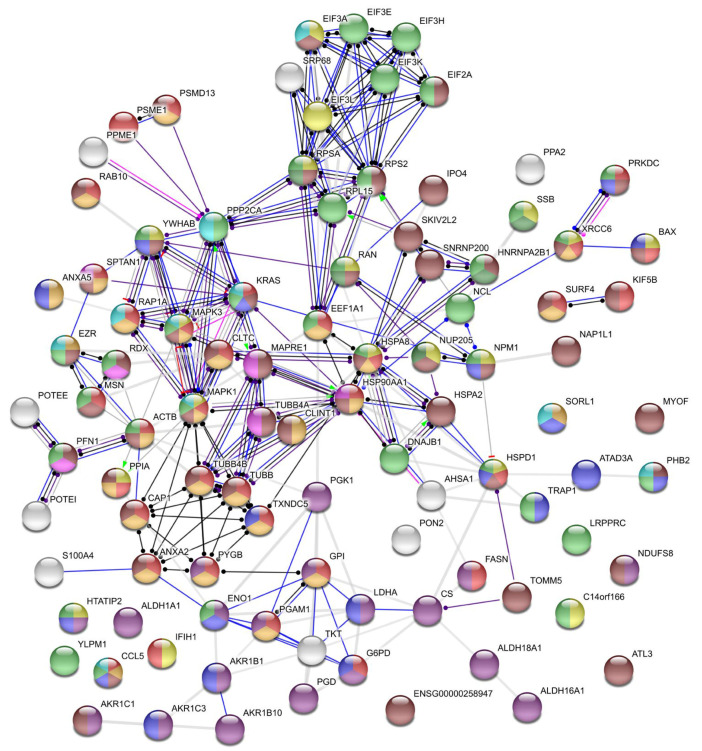
Interaction between proteins putatively mediating binase antiviral activity in IAV-infected cells as generated by STRING. Proteins are represented as nodes, and their functional links are defined by solid lines at high confidence (interaction score above 0.7). Circles are colored according to biological process gene ontology: yellow—viral process (GO:0016032), red—immune system process (GO:0002376), blue—the regulation of cell death (GO:0010941), green—ribonucloprotein complex export from the nucleus (GO:0071426), pink—the regulation of protein polymerization (GO:0032271), light blue—regulation of the MAPK cascade (GO:0043408), light green—the regulation of gene expression (GO:0010468), orange—vesicle-mediated transport (GO:0016192), purple—oxidation-reduction process (GO:0055114), and brown—cellular component organization or biogenesis (GO:0071840). Abbreviations of the proteins are explained in Table S2.

According to the biological process enrichment, proteins involved in the metabolism of cofactors, carbohydrate derivatives, retinoids and lipids, or mediating response to stimulus were increased by binase independently of the treatment scheme (Figure 5 and Table 1). Additionally, under the PI scheme, proteins belonging to membrane docking and immune response were upregulated by binase (Figure 5 and Table 1). Independently of the treatment scheme, in contrast, ATL3, EIF3A, EIF3L, HSPD1, KRAS, LRPPRC, NCL, NPM1, PPA2, RAB10, and TXNDC5 proteins were decreased by binase in VB vs. V cells. Most of them belong to virus-associated processes and endoplasmic reticulum organization (Table 1). Proteins that were additionally downregulated under the preincubation scheme could be attributed to the negative regulation of apoptosis (Figure 5 and Table 1). Under the w/o PI scheme, proteins additionally downregulated by binase in virus-infected cells were united by the following biological processes: establishment of the endothelial barrier, translational initiation, negative regulation of protein polymerization, noncoding (nc)RNA processing, leukocyte and neutrophil activation, symbiont process, nucleocytoplasmic transport, regulation of the ERK1 and ERK2 cascade, gene expression and its regulation, and the establishment of localization (Figure 5 and Table 1).

These results indicate that the antiviral activity of binase against IAV is expressed on the proteomic level by the inversion of virus-induced proteomic changes resulting in the inhibition of virus-associated processes, in the stimulation of the immune response, and in the upregulation of proteins that were downregulated by IAV in the virus-infected cells.

## 3. Discussion

IAV induces complex cellular responses in the infected host cell. Host cell proteins play an essential role in viral propagation. Proteins that are differentially expressed in cells upon IAV infection have been extensively studied to better understand the host response to virus infection [37,38,39]. A comparative data analysis is complicated by the diversity of proteome profiles of virus-infected cells and strongly depends on the virus strain, type of infected cells, and time point of infection [36,39,40]. To our knowledge, proteomic studies involving cell treatment by binase, as well as any other ribonuclease, were not conducted before.

Binase is a small (12 kDa) cationic bacterial ribonuclease with antiviral activity. The molecular basis for this effect is not clearly understood. Binase cleaves phosphodiester bonds in RNA and oligoribonucleotides preferentially after guanyl bases with the formation of 2′,3′-cyclic ribonucleotide derivatives in the first step. Cyclic ribonucleotides are hydrolyzed by binase at a lower rate; therefore, they are present in the mixture for a while [41] and can exert some regulatory effects [42]. Previous studies have demonstrated an inhibition of cellular RNA synthesis by 16% and viral RNA synthesis by 51% in binase-treated cell cultures [26]. However, binase-induced RNA decreases did not correlate with apoptosis in different cell lines [43]. In addition to the breakdown of available RNA, binase was shown to interact with cellular proteins affecting cell-signaling pathways [44].

The binase treatment, as well as viral infection, is a stimulus inducing a cell stress response. In our work, the majority of proteins affected by IAV and binase in A549 cells after 6 h of incubation were enriched in biological processes of cellular metabolism, the response to stimulus, biological regulation, intracellular transport, and the regulation of gene expression. Binase predominantly demonstrated an upregulating mode of action, while the majority of proteins affected by IAV were downregulated (Figure 3). It was found that, inside IAV-infected cells, binase counteracted the IAV action, leading to a decrease of the virus titer (Figure 1), which was concomitant with the restoration in the amount of many proteins to the level of mock-treated cells (Table S2, Figure 3 and Figure 4).

Binase suppressed some proteins associated with viral process (Table S2, Figure 3 and Figure 4). Thus, eukaryotic translation initiation factors EIF3A and EIF3L involved in viral translational termination-reinitiation were decreased in virus-infected cells upon binase treatment. Chaperonin HSPD1 is crucial for IAV replication; it interacts with the viral PB2 protein, facilitating its translocation to the mitochondrion, where PB2 maintains the mitochondrial stability and modulates the host immune response [45]. In our work, the level of HSPD1 was increased in virus-infected cells, (PI) and binase strongly downregulated it (Figure 4**).** Peptidylprolyl isomerase A (PPIA) interacts with protein M1 of IAV and inhibits viral replication [46]. PPIA depletion results in increased IAV infectivity [47]. In virus-infected cells, a drop in the PPIA amount was registered (Figure 3). Binase upregulated PPIA in all binase-treated cells. Under the PI treatment scheme, the level of thioredoxin TXNDC5 was significantly upregulated by the virus (V), whereas the binase treatment of IAV-infected A549 cells (VB) decreased its amount. This could contribute to the antiviral activity of binase, since antioxidant enzymes are important for the IAV life cycle, and their silencing inhibits virus propagation [48]. Moreover, proteins mediating the immune response were also affected. For example, upon PI, binase increases the level of MDA5 (IFIH1, interferon induced with helicase C domain 1), which was suppressed by IAV. It was shown that MDA5 depletion significantly enhances virus replication [49]. Therefore, its increase under binase treatment will contribute to the host antiviral response.

In IAV-infected cells, apoptosis is regulated by the virus. At the early stages of the viral life cycle, apoptosis is inhibited to support viral propagation, and at the late stages, it is activated for the same purpose—to support viral replication [50,51]. In regard to IAV-induced signaling, we also found that binase is able to decrease the level of KRAS and RAP1A, a Ras-related protein, in the virus-infected cells. It is known that binase directly binds to the KRAS kinase and prevents its activation by guanine exchange factor SOS, which leads to the inhibition of Raf/MEK/ERK (MAPK) signaling and cell proliferation [44]. In A549 cells, binase suppresses prohibitin PHB2 (Figure 4), which plays an indispensable role in the activation of the MAPK pathway [52]. Under the w/o PI treatment, the level of PHB2 was also decreased in virus-infected binase-treated cells. The depletion of prohibitins had a negative impact on the phosphorylation of ERK1/2 [52]; targeting prohibitins is regarded as a promising strategy for cancer treatment [53]. Besides, binase has the potential to additionally increase the levels of two negative regulators of the MAPK cascade, SORL1 and PPP2CA, which were upregulated in binase-treated cells (Table S2). Since the MAPK pathway is used by IAV to support its efficient replication by promoting the nuclear export of viral ribonucleoprotein (vRNP) complexes, an inhibition of the MAPK pathway by binase could lead not only to the inhibition of cell proliferation and induction of apoptosis but could also result in a reduction of infectious progeny virions [54].

Proteins participating in the RNP export from the nucleus were suppressed by binase in IAV-infected cells (Figure 5). Nucleolin NCL, which is required for IAV replication and the efficient export of vRNPs [55], was upregulated by IAV under the PI treatment scheme and downregulated by binase in IAV-infected cells under both PI and w/o PI schemes (Figure 4). Nucleolin shuttles between the nucleus and cytoplasm and even translocates to the cell surface [56]. The latter process requires an intact actin cytoskeleton. Nucleolin is able to bind IAV hemagglutinin, facilitating IAV internalization [57]. Additionally, nucleophosmin NPM1, which presumably associates with IAV polymerase and mediates vRNP nuclear export [58], was decreased in IAV-infected cells upon binase treatment (Figure 4). It was found that ribosomal protein RPL5, which associates with IAV vRNP and requires NPM1 for nuclear export [59], and NPM1 knockdown greatly reduce IAV NP accumulation [60]. Under the w/o PI scheme, the level of nucleoporin Nup205, a component of the nuclear pore complex upregulated by IAV [61], was also decreased in VB vs. V cells (Figure 4). The retention of IAV NP within the nucleus is also caused by the knocking out of the BAX protein [62,63]. BAX was found to be reliably decreased in virus-infected binase-treated A549 cells, although only in the experiment without the preincubation of IAV with binase (Table S2). Moreover, binase increases the level of heterogeneous nuclear ribonucleoprotein A2/B1 (HNRNPA2B1), which inhibits virus replication by interacting with the IAV protein NS1 and by suppressing NS1 mRNA nuclear export [64], in V vs. M cells (PI and w/o PI) and in VB vs. V cells (w/o PI) (Figure 4). Thus, nuclear export, which is required for IAV at the stage of 6 h p.i., is affected by binase possibly adding to its antiviral activity. However, nuclear import could be affected as well.

IAV induces cytoskeleton remodeling to support its viral life cycle [65]. The great changes observed in the protein amounts of virus-infected A549 cells were related to cytoskeleton organization. Interestingly, binase treatment interferes with this. The IAV infection decreased the level of several tubulins and intermediate filaments (RDX), but also, the negative regulators of actin polymerization (PFN1, HSPA8, RDX, TUBB, and RAN) were reduced in the infected cell, suggesting that the formation/stabilization of actin fibers in virus-infected cells at 6 h p.i. is important. At this time point p.i., these changes might be related to vRNP transport to the cell membrane along actin fibers and to the formation of progeny virions as the actin-myosin network is important for the assembly of viral particles at the plasma membrane [66]. The viral NP protein binds to filamentous actin late in infection, which leads to the cytoplasmic retention of NP preventing the nuclear reimport of exported vRNPs destined for packaging into progeny virions at the cell membrane [67]. Notably, a reduction of actin fibers in virus-infected cells treated with the anti-inflammatory drug simvastin prevented virion formation [68]. Actin filaments are also involved in the production of filamentous virions and the direct cell-to-cell transport of viral vRNPs, circumventing the need for viral budding from the cell membrane and virus entry into a new cell [69]. This leads to a more rapid dissemination of the virus, allowing evasion from immune- and antiviral defenses. Inhibitors of actin polymerization also attenuate the formation of tunneling nanotubes, long membranous actin-based extensions that connect one cell to another and allow the transfer of viral genomes. At low concentrations, profilin-1 stimulates actin fiber formation, while, at high concentrations, it prevents polymerization. We found that the binase treatment of IAV-infected cells (VB) elevated the level of PFN1, TUBB, and RDX, especially upon the PI scheme of cell treatment, which might impair actin polymerization and, thus, virus propagation. However, whether the disruption of cytoskeletal elements has a dominant effect is not clear, as others have only observed a minimal-to-modest effect on the overall infectious particle assembly process [65].

Therefore, according to our study, binase, in addition to its ability to degrade IAV RNAs within the infected cells, might interfere with vRNP transport for viral particle assembly during IAV infection; however, other aspects of its activity within A549 cells, such as modulating the immune response, could be involved as well. Overall, it seems possible that the binase treatment exerts its antiviral effect in multiple ways. Therefore, further studies are needed that will analyze the roles of the affected cellular proteins in the antiviral mode of action of binase against IAV infection.

## 4. Materials and Methods

### 4.1. Materials and Methods Cell Culture, Virus, and Binase

Human lung adenocarcinoma epithelial A549 cells and Madin-Darby Canine Kidney (MDCK-II) cells (ATCC, Manassas, VA, USA) were grown in 6-well plates in Dulbecco’s modified Eagle’s medium (DMEM) (Gibco, Waltham, MA, USA) supplemented with 10% fetal bovine serum (PAA Laboratories GmbH, Goetzis, Austria Austria), 100 U/mL penicillin, and 0.1 mg/mL streptomycin (P/S) (Gibco, Waltham, MA, USA) at 37 °C in a 5% CO_2_ atmosphere and at 95% humidity. The pandemic influenza A/Hamburg/04/09 (H1N1pdm09) provided by the strain collection of the Institute of Medical Virology, Justus Liebig University, Giessen, Germany was propagated in MDCK-II cells in infection medium (DMEM containing 0.2% bovine albumin (BA) (PAA Laboratories GmbH, Cölbe, Germany), P/S, and 1 μg/mL TPCK-treated trypsin (Sigma-Aldrich; Merck KGaA, Darmstadt, Germany)) at 37 °C, 5% CO_2_, and 95% humidity. Binase was purified from *Bacillus pumilus* 7P culture fluid according to the procedure described in [70]. RNase activity of purified binase was 1.2 × 10^6^ units per milligram of protein.

### 4.2. Determination of Virus Titers by Focus Forming Assay (FFA)

A549 cells were grown for 24 h until 95–100% confluency, as described above. The cells were washed twice with PBS^++^/BA/P/S (phosphate-buffered saline containing 0.2% BA, 1 mM MgCl_2_, 0.9 mM CaCl_2_, and P/S) and infected by the virus (500 μL/well) at a multiplicity of infection of 1 (MOI = 1) that was preincubated either with binase (10 and 100 μg/mL) or PBS for 30 min at room temperature (RT) in the dark. Cell were infected for 1 h at RT in the dark, washed with PBS, and incubated for 8 h. Afterwards, cell supernatants were collected to determine the virus titer in the A549 cell culture after a single-cycle replication of the H1N1pdm09 virus by the focus forming assay (FFA) using MDCK-II cells.

For this, MDCK-II cells were grown in a 96-well plate for 24 h until about 95% confluency. The cells were washed twice with PBS^++^/BA/P/S. Supernatants from A549 cells were serially diluted with PBS and added to the MDCK-II cells (50 μL/well). The MDCK-II cells were incubated for 1 h at RT in the dark. Then, cells were washed with PBS, overlaid by Avicel-containing media (minimum essential medium (Gibco, Waltham, MA, USA) supplemented with 1.25% microcrystalline cellulose (FMC, Belgium), 0.36% BA, P/S, 0.3% NaHCO_3_, 0.01% DEAE-Dextran, and 1 μg/mL TPCK-treated Trypsin) and incubated for 28 h. Then, Avicel-containing overlay media was removed, and cells were washed carefully three to five times with PBS^++^ (PBS containing 1 mM MgCl_2_ and 0.9 mM CaCl_2_).

Cells were fixed with 150 μL of fixing solution containing 3.7% paraformaldehyde (PFA) (Roth, Karlsruhe, Germany) and permeabilized by 1% Triton X-100 (Roth, Karlsruhe, Germany) diluted in PBS^++^ during 45 min at RT. Cells were washed three times with PBS containing 0.05% Tween 20 (Sigma-Aldrich; Merck KGaA, Darmstadt, Germany) (PBST) and incubated with 50 μL/well of mouse anti-IAV NP primary antibody diluted 1:100 in PBS containing 3% bovine serum albumin (BSA) (Sigma-Aldrich; Merck KGaA, Darmstadt, Germany) for 1.5 h at RT. Then, cells were washed three times with PBST and consequently incubated with 50 μL/well of anti-mouse horseradish peroxidase-coupled secondary antibody (Santa Cruz Biotechnology, Dallas, TX, USA) diluted 1:1000 in PBS containing 3% BSA for 1 h at RT in the dark, followed by three-times washing with PBST. Cells were stained by AEC (3-amino-9-ethylcarbazole) staining solution (40 μL/well) for 45 min at 37 °C. Finally, cells were washed with double-distilled water (ddH_2_O) and dried. The 96-well plate was scanned using the Epson Perfection V500 photo scanner (Seiko Epson Corporation, Japan). Quantity of foci in each well was calculated using Photoshop software package (ver. 8; Adobe, San Jose, CA, USA). Results represent the mean from three independent biological and two technical replicates.

### 4.3. Cell Treatment and Lysis

A549 cells grown for 24 h until about 100% confluency were washed twice with PBS^++^/BA/P/S. Afterwards, cells were treated in four different ways: mock-treated (M), treated by 100 μg/mL binase (B), infected by virus at a multiplicity of infection of 3 (MOI = 3) (V), and virus-infected binase-treated at the same concentrations (VB).

Two schemes of cell treatments, including and not including a 30 min preincubation of the virus with binase in PBS prior to cell application, were applied. In the preincubation (PI) scheme, the virus, binase, or the mixture of virus and binase were incubated in PBS for 30 min at RT in the dark. Then, cells were treated either with PBS (M), binase (B), virus (V), or the mixture of virus and binase (VB) for 1 h at RT in the dark, washed, and further grown in DMEM (variants M and V) or 100 μg/mL binase-containing DMEM (variants B and VB). The scheme without the preincubation of the virus with binase (w/o PI) included the treatment of cells by the virus or PBS for 1 h at RT in the dark, the wash step, and further growth in DMEM (variants M and V) or 100 μg/mL binase-containing DMEM (variants B and VB).

After 6 h, cells were washed twice with PBS, left on ice in 1 mL ice-cold PBS for 5 min, and then scrubbed into a microtube and pelleted by centrifugation at 3000× *g* for 5 min at 4 °C. Cell pellets were lysed in lysis buffer (25 mM Tris, pH 8.0, 137 mM NaCl, 10% glycerol, 0.1% sodium dodecyl sulphate, 0.5% sodium deoxycholate, 1% NP-40, 2 mM ethylenediaminetetraacetic acid (EDTA), pH 8.0, 0.2 mM Pefablock, 5 μg/mL aprotinin, 5 μg/mL leupeptin, 1 mM Na-vanadate, and 5 mM benzamidine) for 10 min on ice, incubated in a ultrasonic bath (Elmasonic, Elma Schmidbauer GmbH, Singen, Germany) with ice-cold water for 10 min, and subsequently, centrifuged for 30 min at 13,000× *g* at 4 °C. Supernatants were collected in a fresh ice-cold microtube, immediately stored at −20 °C for 1 h, and then kept at −80 °C until further manipulations. In the experiments, three replicates were used for each sample.

### 4.4. Qualitative Shotgun Proteomics

#### 4.4.1. “In-Solution” Protein Digestion

Proteins were trypsin-digested in the solution according to the procedure described by Erde and colleagues [71], with some modifications. Protein concentrations were measured using a BCA (bicinchoninic acid) protein assay kit (Merck, Kenilworth, NJ, USA). Cell lysate supernatants with a protein amount of 300 μg were incubated in the presence of 0.1M dithiothreitol (DTT) for 30 min at 60 °C. The volume of each sample was adjusted to a total volume of 300 μL with urea-containing ammonium bicarbonate buffer (UABB; 100 mM ammonium bicarbonate, 8 M urea, 0.1% sodium deoxycholate, and 0.1% octyl glucoside). Then, samples were centrifuged using an Amicon Ultra-0.5 10-kDa molecular weight cut-off centrifugal filter unit (Merck, Kenilworth, NJ, USA) for 20 min at. Then, filters were washed twice with UABB. The reduced cysteine residues were alkylated with 35 mM iodoacetamide in UABB at RT in the dark for 1 h. Samples were centrifuged for 20 min at 14,000× *g*. Residual iodoacetamide was neutralized with 162 mM DTT in UABB. The filters were washed once with UABB and three times with urea-free ammonium bicarbonate buffer (ABB). Then, 50 µL of ABB containing 6 µg of trypsin (1:50) were added. After incubation for 16 h at 37 °C, samples were centrifuged for 20 min at 14,000× *g*, and filtrates were collected. The residual peptides were washed out twice with 50 μL of ABB, and filtrates were combined. The pH was adjusted to 4.0 with 10% trifluoroacetic acid. To get rid of detergent sediments, the samples were centrifuged for 10 min at 14,000× *g*. Peptide solutions were purified using Discovery DSC-18 cartridges for solid-phase extraction (Merck, Kenilworth, NJ, USA).

#### 4.4.2. LC-MS/MS Analysis

Liquid chromatography–tandem mass spectrometry (LC-MS/MS) was performed using a chromatography system Dionex Ultimate 3000 (Thermo Scientific Dionex, Waltham, MA, USA) coupled to a maXis Impact mass spectrometer (Bruker Daltonik GmbH, Bremen, Germany) with a Captive Spray electrospray ionization platform. Trypsin-digested peptides were separated using an AcclaimPepMap C18 column (2 µm, 100 Å, 75 µm × 15 cm; Thermo Scientific, Waltham, MA, USA). The mobile phases consisted of 0.1% formic acid and 5% acetonitrile in water (solvent A) and 0.1% formic acid in 94.9% acetonitrile (solvent B). Chromatographic separation was performed in several steps: 0–5 min, 2% solvent B; 5–160 min, gradient from 2 to 55% solvent B; 160–165 min, gradient from 55 to 90% solvent B; 165–180 min, 90% solvent B; 180–190 min, gradient from 90 to 2% solvent B; and 190–195 min, 2% solvent B. The flow rate was 300 nL/min. The separation temperature was 40 °C. A spectrum of positively charged ions was obtained using the following settings: 3 L/min gas flow rate, 150 °C temperature, 1600 V voltage, a detection range of 50–2200 m/z, 10 Hz sampling frequency, and automatic ion fragmentation in MS/MS mode.

#### 4.4.3. Database Search

Mass spectra were processed using DataAnalysis 4.1. Proteins were identified using the MASCOT search engine (Version 2.4.0; Matrix Science, Boston, MA, USA) against the *Homo sapiens* Swiss-Prot database using the following search criteria: a maximum of one missed trypsin cleavage, a fixed carbamidomethyl modification of cysteines, and variable methionine oxidation, with 50 ppm peptide mass tolerance (0.6 Da mass error tolerance). Proteins were identified using at least two peptides, with the MASCOT acceptance score being above the threshold of 1% probability that protein identification is incorrect (false discovery rate, FDR). The FDR was calculated based on peptide-spectrum matches using the Mascot Percolator algorithm. The *p*-value was less than 0.011. The MS proteomics data were deposited in the ProteomeXchange Consortium via the jPOSTrepo (Japan ProteOme STandard Repository) with the dataset identifier PXD013545 [72].

### 4.5. Quantitative Targeted Proteomics

#### 4.5.1. “In-gel” Protein Digestion

For the relative quantitative analysis, 200 μg of total proteins from cell lysates were loaded onto 16% polyacrylamide gel. Electrophoresis was carried out until proteins entered the separating gel and ran 1.5–2 cm. Gels were stained with a Coomassie G-250. Stained bands were cut out and divided into 1 × 1 × 1-mm cubes. The dye was washed from gel pieces, and these were then dehydrated with acetonitrile. Proteins were digested by the addition of trypsin (1 μg of the enzyme per 60 μg of proteins in 100 mM ammonium carbonate with 10% acetonitrile) at 37 °C for 16 h. Peptides were then extracted using ultrasound.

#### 4.5.2. “In-Silico” Protein Digestion

In-silico protein digestion of proteins under interest was performed in Skyline software (ver. 4.2.0.19072; Skyline Software Systems, Herndon, VA, USA). Peptide settings were as follows: trypsin enzyme, peptide length 6–18 amino acids (aa)-long, none missed cleavages. For fragment selection, peptides that contained cysteine and methionine were excluded from the analysis because of their possible modification. For multiple reaction monitoring (MRM) analysis, the following transition settings were used: precursor charges, 2; fragment ion charges, 1; ion type, y or b; and product ions, 3. Generated peptides were checked by “blastp” (Refseq database, organism Homo sapiens), and only peptides unique for each protein were retained. The time of protein retention on the column was predicted based on the elution of iRT (indexed Retention Time) standards.

#### 4.5.3. Targeted LC-MS/MS Analysis (LC-MRM/MS)

Peptide detection was carried out using the MRM method on a QTRAP 6500+ LC-MS/MS system (AB Sciex, Foster City, CA, USA) combined with a 1290 Infinity II LC system (Agilent, Santa Clara, CA, USA). Trypsin-digested peptides were separated using a Titan C18 column (1.9 µm, 10 cm × 2.1 mm; Supelco, Bellefonte, PA, USA). The mobile phases consisted of 0.1% formic acid and 5% acetonitrile in water (solvent A) and 0.1% formic acid in 94.9% acetonitrile (solvent B). Chromatographic separation was performed on several steps: 0–0.1 min, gradient from 3 to 10% solvent B; 0.1–3 min, gradient from 10 to 11% solvent B; 3–13 min, gradient from 11 to 19% solvent B; 13–13.5 min, gradient from 19 to 20% solvent B; 13.5–13.6 min, gradient from 20 to 23% solvent B; 13.6–16.7 min, gradient from 23 to 25% solvent B; 16.7–18.5 min, gradient from 25 to 95% solvent B; 18.5–21.9 min, 95% solvent B; 21.9–23 min, gradient from 95 to 3% solvent B; and 23–25 min, 3% solvent B. The flow rate was 0.4 mL/min. The separation temperature was 40 °C. The injection volume was 5 μL. A spectrum of positively charged ions was detected by an IonDrive V Turbo Spray source (AB Sciex, Foster City, CA, USA) in scheduled MRM (multiple reaction monitoring) mode using the following settings: 500 °C source temperature, 5200 V ion spray voltage, 35 psi curtain gas pressure, 60 psi nebulizer gas pressure, and 60 psi auxiliary gas pressure. Collision energy and declustering potential was calculated for each peptide using Skyline software (ver. 4.2.0.19072; Skyline Software Systems, Herndon, VA, USA). At least two peptides per protein were detected. The representation of proteins in the sample was evaluated using the MultiQuant software (ver. 3.0.2; SCIEX, Redwood City, CA, USA) by the peak area of MRM transitions specific for each peptide studied. The data were manually inspected to ensure correct peak detection and accurate integration. The MS proteomics data were deposited with the dataset identifier PXD014809 in the ProteomeXchange Consortium via the jPOSTrepo (Japan ProteOme STandard Repository) (Okuda et al., 2017).

### 4.6. Protein Networks and Pathway Analysis

Protein classification according to the biological process, molecular function, and cellular component was performed using gene ontology (GO) by the PANTHER (Protein ANalysis THrough Evolutionary Relationships) classification system (http://pantherdb.org). To predict protein-protein interactions, the STRING (Search Tool for the Retrieval of Interacting Genes/Proteins) database v11.0 was used [73]. The bonds between nodes were calculated with a high confidence score (0.7).

### 4.7. Statistical Analysis

All experiments were performed in three biological repeats and two technical replicates in FFA or three technical replicates in LC-MRM/MS. Statistical tests and graphical data presentation were performed using Microsoft Excel 2010 (Microsoft, Redmond, WA, USA) and GraphPad Prism software ver. 5.01 (GraphPad Software, San Diego, CA, USA). Data are presented as the mean ± standard deviation of the mean. The significance between two groups in FFA (100 μg/mL binase-treated vs. nontreated) was determined via Student’s *t*-test. The significance between four groups (M, B, V, and VB) and two schemes of cell treatment (PI and w/o PI) was analyzed by a two-way analysis of variance (ANOVA) and the Bonferroni post-hoc test. Statistical significance was defined as * *p* < 0.05, ** *p* < 0.01, and *** *p* < 0.001.

## Figures and Tables

**Table 1:** Enrichment of A549 cell proteins affected by the virus and/or binase in the gene ontology (GO) biological processes. IAV: influenza A virus.

Binase or IAV Effect	GO Term	Biological Process or Cellular Component	Proteins
Downregulated by IAV			
	GO:0051186	Cofactor metabolic process	PKM, ENO1, PGK1, LDHA, GPI, PGAM, G6PD, TKT, AKR1B1, AKR1B10, AKR1C3, AKR1C1, HSP90AA1
	GO:0044281	Small-molecule metabolic process	SORL1, ALDH1A1, ACSS3, RAN, HSPA8, TARS, MAPK1, MAPK3
	GO:0006955	Immune response	CCL5, MDA5, PPIA, HSPA8, HSP90AA1, EEF1A1, MAPK1, MAPK3, TUBB4B, TUBB, PKM, ACTB, GPI, PGAM1, G6PD
	GO:0007010	cytoskeleton organization	TUBB3, TUBA4A, TUBB4B, ACTB, PFN1
	GO:0006810, GO:0051179	transport and localization	SORL1, RDX, RAN, YWHAB, TOMM5
Upregulated by IAV			
	GO:0045321	leucocyte activation	SPTAN1, DDX3X, HSPD1, RAB10, ANXA2, TXNDC5
	GO:0065003	protein-containing complex assembly	ATL3, EIF3A, NDUFS8, ANXA2, HSPD1, DDX3X
Downregulated by binase			
	GO:0016020	Membrane	LRPPRC, TRAP1, HSPD1, PHB2, SURF4, NDUFS8, KRT10, PON2, MYOF, RAP1A
Upregulated by binase			
	GO:0010468	regulation of gene expression	PFN1, DNAJB1, EEF1A1, PPP2CA, PRKDC, XRCC6, NCL, RDX, MSN, ACTB, MAPK1, MAPK2, YWHAB, HSPA8, RAN, RPS2, RPSA, RPL15, EIF3K, EIF2A, EIF3E, EIF3H, HNRNPA2B1, CAND1, CCL5, TRAP1, C14ORF166, ENO1
	GO:0006887	exocytosis	CAND1, CCL5, PGAM1, GPI, PKM, ANXA2, PYGB, CAP1, TUBB, TUBB4B, TUBB4A, TXNDC5, PPI, MAPK1, RAP1A, HSP90AA1, HSPA8, ANXA5, EEF1A1, SPTAN, PSMD13, XRCC6
	GO:0006897	endocytosis	MAPK3, MAPK1, HSP90AA1, CLTC, ACTB, CAP1, SORL1, TXNDC5
	GO:0051641	cellular localization	RAN, IPO4, HNRNPA2B1, HNRNPA1L2, KIF5B, C14orf166, RDX, MSN, SRP68, RPSA, RPS2, RPL25, BAX, YWHAB, TUBB4A, TUBB, TUBB4B, ACTB, CLTC, ANXA2, ANXA5, SORL1, AKR1C1, AKR1C3, TXNDC5, PPIA
	GO:0042786	immune process	MDA5, CCL5, PGAM1, PKM, HSPA8, PSMD3, CAND1, KIF5B, XRCC6, RAP1A, HSP90AA1, EEE1A1, MAPK1, PPIA, GPI, PYGB, CAP1, ANXA2, SPTAN1, BAX, TUBB4B, TXNDC5, TUBB, MSN
	GO:0055114, GO:0008152	oxidation-reduction and metabolic processes	AKR1C3, AKR1C1, AKR1B10, ALDH16A1, ALDH18A1, ALDH1A1, ALDH3A1, FASN, PYGB, GPI, G6PD, LDHA, ENO1, PGAM1, PKM, PGK1
Upregulated by binase in IAV-infected cells			
	GO:0051186, GO:1901135, GO:0001523, GO:0006629, GO:0050896	cofactor metabolic process, carbohydrate derivative metabolic process, retinoid metabolic process, lipid metabolic process, response to stimulus	AKR1B1, AKR1B10, AKR1C1, AKR1C3, ANXA5, ENO1, PFN1, PGAM1, PGK1, PPIA
	GO:0022406	membrane docking	TUBB, TUBB4A, TUBB4B, HSP90AA1
	GO:0006955	immune response	MDA5, CCL5, CAP1, KIF5B, MAPK3, TUBB, TUBB4A, TUBB4B, HSP90AA1
Downregulated by binase in IAV-infected cells			
	GO:0016032	viral process	NPM1, HSPD1, EIF3L, EIF3A
	GO:0007029	endoplasmic reticulum organization	RAB10, ATL3
	GO:0043066	negative regulation of apoptosis	TXNDC5, KRAS, NPM1, HSPD1, and HTATIP2
	GO:0061028	establishment of endothelial barrier	RDX, EZR, RAP1A, FASN
	GO:0006413	translational initiation	RPL15, RPSA, EIF3H, EIF3E, RPS2
	GO:0032272	negative regulation of protein polymerization	MAPRE1, SRTAN1, RDX
	GO:0034470	ncRNA processing	SSB, C14orf166, RPSA, RPS2, SKIV2L2
	GO:0045321, GO:0042119	leukocyte and neutrophil activation	BAX, CCL5, SPTAN1, RAP1A, CAP1, SURF4, PRKDC
	GO:0044056	symbiont process	CCL5, NUP205, BAX, C14orf166, RPSA, SSB
	GO:0006913	nucleocytoplasmic transport	PHB2, RPSA, NUP205, SSB
	GO:0070372	regulation of ERK1 and ERK2 cascade	PHB2, RAP1A, CCL5, EZR
	GO:0010467, GO:0006349	gene expression and its regulation	PRKDC, TRAP1, DNAJB1, YLPM1, C14orf166, SSB, RDX, EZR, CCL5, RPSA, RPL15, RPS2, EIF3H, EIF3E, PHB2, SNRNP200, SKIV2L2, BAX, NUP205
	GO:0051234	establishment of localization	CLINT1, CCL5, NUP205, BAX, MAPRE1, C14orf166, RPL15, RPSA, RPS2, SSB, RAP1A, SPTAN1, EZR, RDX, CAP1, SURF4, PHB2

## Supplementary Materials

Supplementary Materials can be found at https://www.mdpi.com/1422-0067/21/21/8294/s1. Table S1: Abundance of proteins affected by virus and/or binase in mock-treated (M), binase-treated (B), virus-infected (V) and virus-infected binase-treated (VB) samples. The blue and white colors indicate that the respective protein was either detected or not detected, respectively, in the sample above 1% FDR cut-off. Table S2: Abundance of proteins affected by virus and/or binase in mock-treated (M), binase-treated (B), virus-infected (V) and virus-infected binase-treated (VB) samples. The red and green colors indicate that the respective protein was either increased or decreased, respectively, in the sample as compared to mock-treated cells (*p*-Val ≤ 0.05). Asterisks distinguish proteins that were significantly (*p*-Val ≤ 0.05) changed in VB samples as compared to V sample suggesting antiviral potential.
