# Supplementary material for: Anti-Influenza Activity of the Ribonuclease Binase: Cellular Targets Detected by Quantitative Proteomics
Source: Int J Mol Sci. 2020 Nov 5;21(21):8294. doi: 10.3390/ijms21218294 (PMC7663932; doi:10.3390/ijms21218294)
Supplement: Supplementary file 1 [file ijms-21-08294-s001.pdf]

Table S1. Abundance of proteins affected by the virus and/or binase in mock-treated (M), binase-treated (B), virus-infected (V), and virus-infected binase-treated (VB) samples. The blue and white colors indicate that the respective protein was either detected or not detected, respectively, in the sample above 1% FDR cut-off.

Protein	Mock	Binase	Virus	Virus+Binase	Description
ACTB					Actin, cytoplasmic 1
ATL2					Atlastin-2
CCT7					T-complex protein 1 subunit eta
CS					Citrate synthase, mitochondrial
DES					Desmin
EEF1A1					Elongation factor 1-alpha 1
EFTUD2					116 kDa U5 small nuclear ribonucleoprotein component
ENO1					Alpha-enolase
EZR					Ezrin
GPI					Glucose-6-phosphate isomerase
HIST1H2BB					Histone H2B type 1-B
HNRNPA2B1					Heterogeneous nuclear ribonucleoproteins A2/B1
HSPD1					60 kDa heat shock protein, mitochondrial
KRT75					Keratin, type II cytoskeletal 75
LRPPRC					Leucine-rich PPR motif-containing protein, mitochondrial
NAP1L1					Nucleosome assembly protein 1-like 1
NCL					Nucleolin
NDUFS8					NADH dehydrogenase [ubiquinone] iron-sulfur protein 8, mitochondrial
PGK1					Phosphoglycerate kinase 1
POTEE					POTE ankyrin domain family member E
POTEI					POTE ankyrin domain family member I
PRPH					Peripherin
RAP1A					Ras-related protein Rap-1A
RPS2					40S ribosomal protein S2
SF3B1					Splicing factor 3B subunit 1
TALDO1					Transaldolase
TARS					Threonine--tRNA ligase, cytoplasmic
TARSL2					Probable threonine--tRNA ligase 2, cytoplasmic
TKT					Transketolase
AHSA1					Activator of 90 kDa heat shock protein ATPase homolog 1
HSPA2					Heat shock-related 70 kDa protein 2
RPL15					60S ribosomal protein L15
TXNDC5					Thioredoxin domain-containing protein 5
DDX18					ATP-dependent RNA helicase DDX18

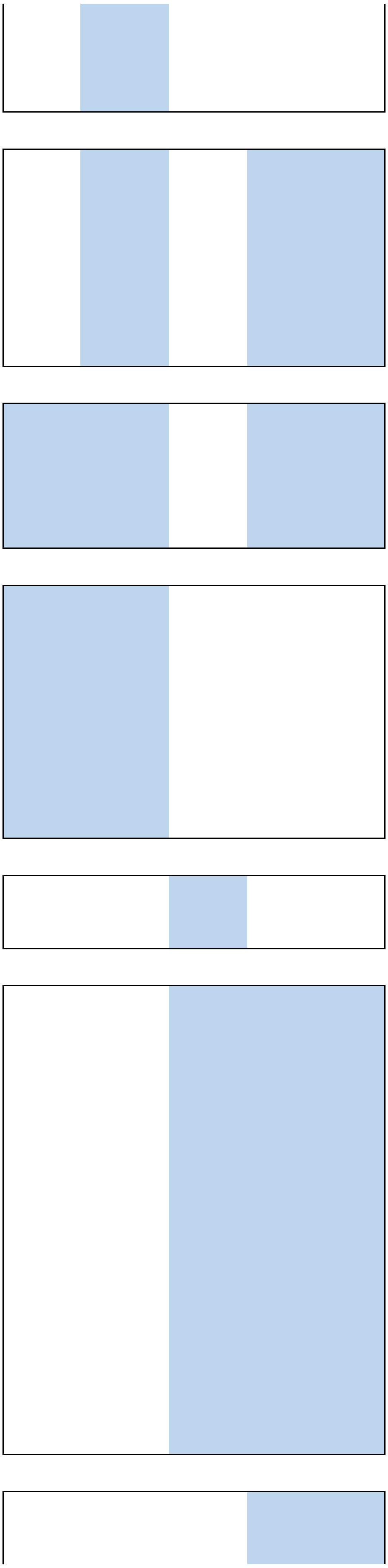

DNAJB1	DnaJ homolog subfamily B member 1
STXBP2	Syntaxin-binding protein 2
TOMM7	Mitochondrial import receptor subunit TOM7 homolog
CLINT1	Clathrin interactor 1
EIF3A	Eukaryotic translation initiation factor 3 subunit A
KIF5B	Kinesin-1 heavy chain
MARS	Methionine--tRNA ligase, cytoplasmic
PON2	Serum paraoxonase/arylesterase 2
PYGB	Glycogen phosphorylase, brain form
AKR1C1	Aldo-keto reductase family 1 member C1
CLTCL1	Clathrin heavy chain 2
PFN1	Profilin-1
POTEKP	Putative beta-actin-like protein 3
HSPA8	Heat shock cognate 71 kDa protein
LDHA	L-lactate dehydrogenase A chain
PGAM1	Phosphoglycerate mutase 1
TBB8L	Tubulin beta-8 chain-like protein LOC260334
TUBB3	Tubulin beta-3 chain
TUBB4A	Tubulin beta-4A chain
VIM	Vimentin
MAPRE1	Microtubule-associated protein RP/EB family member 1
PPP1R14B	Protein phosphatase 1 regulatory subunit 14B
AKT1	RAC-alpha serine/threonine-protein kinase
ALDH16A1	Aldehyde dehydrogenase family 16 member A1
ATAD3A	ATPase family AAA domain-containing protein 3A
CMSS1	Cms1 Ribosomal Small Subunit Homolog
EHD2	EH domain-containing protein 2
EIF3L	Eukaryotic translation initiation factor 3 subunit L
FAF2	FAS-associated factor 2
GNPAT	Dihydroxyacetone phosphate acyltransferase
GOLPH3L	Golgi phosphoprotein 3-like
IPO4	Importin-4
MYO1C	Unconventional myosin-Ic
PRKDC	DNA-dependent protein kinase catalytic subunit
TOMM5	Mitochondrial import receptor subunit TOM5 homolog
ALDH18A1	Delta-1-pyrroline-5-carboxylate synthase
ATL3	Atlastin-3

BAX	Apoptosis regulator BAX
CAND1	Cullin-associated NEDD8-dissociated protein 1
HTATIP2	Oxidoreductase HTATIP2
KDELR2	ER lumen protein-retaining receptor 2
KDM1A	Lysine-specific histone demethylase 1A
MACF1	Microtubule-actin cross-linking factor 1, isoforms 1/2/3/5
MSH2	DNA mismatch repair protein Msh2
NUP205	Nuclear pore complex protein Nup205
PRPF18	Pre-mRNA-splicing factor 18
PRPS1	Ribose-phosphate pyrophosphokinase 1
SRP68	Signal recognition particle subunit SRP68
XRCC6	X-ray repair cross-complementing protein 6
ACTC1	Actin, alpha cardiac muscle 1
AKR1B15	Aldo-keto reductase family 1 member B15
AKR1C2	Aldo-keto reductase family 1 member C2
AP1B1	AP-1 complex subunit beta-1
EEF1A2	Elongation factor 1-alpha 2
EIF1AX	Eukaryotic translation initiation factor 1A, X-chromosomal
EIF3K	Eukaryotic translation initiation factor 3 subunit K
HNRNPA1L2	Heterogeneous nuclear ribonucleoprotein A1-like 2
KRT10	Keratin, type I cytoskeletal 10
MSN	Moesin
NEDD8	Ubiquitin-Like Protein Nedd8
PGD	6-phosphogluconate dehydrogenase, decarboxylating
PPIA	Peptidyl-prolyl cis-trans isomerase A
PSME1	Proteasome activator complex subunit 1
RDX	Radixin
TUBA1A	Tubulin alpha-1A chain
TUBB	Tubulin beta chain
YWHAB	14-3-3 protein beta/alpha

Table S2. Abundance of proteins affected by the virus and/or binase in mock-treated (M), binase-treated (B), virus-infected (V), and virus-infected binase-treated (VB) samples. The red and green colors indicate that the respective protein was either increased or decreased, respectively, in the sample, as compared to mock-treated cells (p-Val ≤ 0.05). Asterisks distinguish proteins that were significantly (p-Val ≤ 0.05) changed in VB samples as compared to V samples, suggesting antiviral potential.

	preincubation					without preincubation					
**Protein**	**M**	**B**	**V**	**VB**		**M**	**B**	**V**	**VB**		**Description**
**AHSA1**	1.1E ± 05	1.3E ± 05	9.4E ± 04	1.1E ± 05	*	1.5E ± 05	1.6E ± 05	1.4E ± 05	1.5E ± 05		Activator of 90 kDa heat shock protein ATPase homolog 1
**AKR1B1**	8.5E ± 05	1.0E ± 06	6.3E ± 05	7.8E ± 05	*	7.3E ± 05	9.1E ± 05	6.7E ± 05	7.8E ± 05	*	Aldo-keto reductase family 1 member B1
**AKR1B10**	3.2E ± 05	4.6E ± 05	2.7E ± 05	3.4E ± 05	*	4.1E ± 05	5.3E ± 05	4.1E ± 05	5.0E ± 05	*	Aldo-keto reductase family 1 member B10
**AKR1C1**	4.9E ± 06	6.2E ± 06	3.6E ± 06	4.6E ± 06	*	4.9E ± 06	6.4E ± 06	4.5E ± 06	5.4E ± 06	*	Aldo-keto reductase family 1 member C1
**ALDH1A1**	2.1E ± 06	3.0E ± 06	1.6E ± 06	2.3E ± 06	*	2.7E ± 06	3.2E ± 06	2.5E ± 06	2.6E ± 06		Retinal dehydrogenase 1
**ANXA5**	3.5E ± 05	5.0E ± 05	2.7E ± 05	3.8E ± 05	*	4.1E ± 05	4.9E ± 05	3.7E ± 05	4.2E ± 05	*	Annexin A5
**CCL5**	1.8E ± 04	2.3E ± 04	1.3E ± 04	1.9E ± 04	*	2.4E ± 04	2.4E ± 04	2.2E ± 04	1.6E ± 04	*	C-C motif chemokine 5
**eEF1A**	1.4E ± 07	1.6E ± 07	1.1E ± 07	1.3E ± 07	*	1.6E ± 07	2.0E ± 07	1.6E ± 07	1.6E ± 07		Elongation factor 1-alpha
**ENO1**	1.3E ± 05	1.8E ± 05	1.1E ± 05	1.4E ± 05	*	1.6E ± 05	2.0E ± 05	1.5E ± 05	1.7E ± 05	*	Alpha-enolase
**ERK1**	1.5E ± 05	1.8E ± 05	1.2E ± 05	1.4E ± 05	*	1.6E ± 05	1.9E ± 05	1.4E ± 05	1.5E ± 05		Mitogen-activated protein kinase 3
**G6PD**	1.9E ± 06	2.4E ± 06	1.7E ± 06	2.0E ± 06	*	2.4E ± 06	2.9E ± 06	2.3E ± 06	2.5E ± 06		Glucose-6-phosphate 1-dehydrogenase
**GPI**	4.6E ± 05	5.8E ± 05	4.0E ± 05	4.6E ± 05	*	5.5E ± 05	6.6E ± 05	5.1E ± 05	5.5E ± 05		Glucose-6-phosphate isomerase
**HSP90AA1**	5.1E ± 05	6.7E ± 05	4.0E ± 05	5.1E ± 05	*	6.4E ± 05	7.3E ± 05	6.0E ± 05	5.9E ± 05		Heat shock protein HSP 90-alpha
**LDHA**	2.7E ± 06	3.2E ± 06	1.9E ± 06	2.7E ± 06	*	3.2E ± 06	3.8E ± 06	2.9E ± 06	3.0E ± 06		L-lactate dehydrogenase A chain
**MDA5**	8.4E ± 04	1.0E ± 05	6.8E ± 04	8.8E ± 04	*	9.7E ± 04	1.2E ± 05	9.3E ± 04	1.0E ± 05		Interferon-induced helicase C domain-containing protein 1
**PFN1**	1.8E ± 06	2.6E ± 06	1.3E ± 06	1.9E ± 06	*	2.9E ± 06	3.5E ± 06	2.8E ± 06	3.1E ± 06	*	Profilin-1
**PGAM1**	2.6E ± 04	3.5E ± 04	2.0E ± 04	2.7E ± 04	*	3.1E ± 04	3.8E ± 04	2.7E ± 04	3.0E ± 04	*	Phosphoglycerate mutase 1
**PGK1**	8.0E ± 05	1.0E ± 06	6.1E ± 05	7.8E ± 05	*	8.5E ± 05	1.1E ± 06	7.6E ± 05	9.4E ± 05	*	Phosphoglycerate kinase 1
**PPP2CA**	1.6E ± 05	1.8E ± 05	1.4E ± 05	1.5E ± 05	*	1.8E ± 05	2.1E ± 05	1.6E ± 05	1.7E ± 05		Serine/threonine-protein phosphatase 2A catalytic subunit alpha isoform

**RAN**	6.0E ± 05	8.1E ± 05	4.3E ± 05	5.9E ± 05	*	7.9E ± 05	9.4E ± 05	7.4E ± 05	6.9E ± 05		GTP-binding nuclear protein Ran
**RDX**	6.3E ± 04	7.1E ± 04	5.4E ± 04	6.6E ± 04	*	6.7E ± 04	7.6E ± 04	6.4E ± 04	5.5E ± 04	*	Radixin
**TKT**	3.5E ± 06	4.4E ± 06	2.8E ± 06	3.6E ± 06	*	4.1E ± 06	4.8E ± 06	3.7E ± 06	4.1E ± 06		Transketolase
**TUBB**	1.1E ± 06	1.4E ± 06	7.8E ± 05	1.0E ± 06	*	1.4E ± 06	1.6E ± 06	1.2E ± 06	1.3E ± 06		Tubulin beta chain
**TUBB3**	5.0E ± 04	6.3E ± 04	3.9E ± 04	4.9E ± 04	*	5.3E ± 04	6.6E ± 04	5.1E ± 04	4.9E ± 04		Tubulin beta-3 chain
**TUBB4B**	4.4E ± 05	5.3E ± 05	3.2E ± 05	4.5E ± 05	*	5.6E ± 05	7.0E ± 05	4.9E ± 05	5.3E ± 05		Tubulin beta-4B chain
**YWHAB**	2.0E ± 05	2.9E ± 05	1.5E ± 05	2.2E ± 05	*	2.7E ± 05	3.1E ± 05	2.6E ± 05	2.7E ± 05		14-3-3 protein beta/alpha
**ACTB**	7.8E ± 06	9.1E ± 06	6.4E ± 06	7.3E ± 06		8.7E ± 06	1.0E ± 07	8.6E ± 06	9.3E ± 06		Actin. cytoplasmic 1
**ALDH3A1**	2.5E ± 05	3.1E ± 05	2.0E ± 05	2.3E ± 05		3.1E ± 05	3.9E ± 05	2.8E ± 05	3.1E ± 05		Aldehyde dehydrogenase, dimeric NADP-preferring
**DNAJB1**	5.4E ± 04	6.2E ± 04	4.8E ± 04	4.9E ± 04		7.2E ± 04	7.7E ± 04	6.5E ± 04	5.6E ± 04	*	DnaJ homolog subfamily B member 1
**ERK2**	8.7E ± 04	1.0E ± 05	7.2E ± 04	8.1E ± 04		9.7E ± 04	1.2E ± 05	8.6E ± 04	8.6E ± 04		Mitogen-activated protein kinase 1
**HSPA8**	5.7E ± 06	6.8E ± 06	5.2E ± 06	5.6E ± 06		6.8E ± 06	7.5E ± 06	6.6E ± 06	6.2E ± 06		Heat shock cognate 71 kDa protein
**PKM2**	4.8E ± 06	5.5E ± 06	4.0E ± 06	4.5E ± 06		5.3E ± 06	6.2E ± 06	5.1E ± 06	5.6E ± 06		Pyruvate kinase PKM
**RPSA**	8.2E ± 05	9.2E ± 05	7.2E ± 05	7.5E ± 05		1.0E ± 06	1.1E ± 06	9.4E ± 05	8.4E ± 05	*	40S ribosomal protein SA
**TARS**	2.9E ± 04	3.6E ± 04	2.4E ± 04	2.7E ± 04		3.8E ± 04	4.2E ± 04	3.4E ± 04	3.1E ± 04		Threonine--tRNA ligase, cytoplasmic
**AKR1C3**	9.9E ± 05	1.2E ± 06	6.8E ± 05	8.2E ± 05	*	9.1E ± 05	1.2E ± 06	8.1E ± 05	9.4E ± 05	*	Aldo-keto reductase family 1 member C3
**S100A4**	7.4E ± 04	9.9E ± 04	3.6E ± 04	6.0E ± 04	*	6.9E ± 04	9.6E ± 04	5.5E ± 04	6.1E ± 04		S100 calcium binding protein A4
**SORL1**	2.4E ± 05	2.8E ± 05	1.6E ± 05	2.1E ± 05	*	2.5E ± 05	3.0E ± 05	2.3E ± 05	2.2E ± 05		Sortilin-related receptor
**PPIA**	9.6E ± 05	1.4E ± 06	8.2E ± 05	1.1E ± 06	*	1.2E ± 06	1.4E ± 06	1.2E ± 06	1.3E ± 06	*	Peptidyl-prolyl cis-trans isomerase A
**HNRNPA2B1**	8.9E ± 05	1.2E ± 06	7.5E ± 05	1.2E ± 06	*	9.5E ± 05	1.2E ± 06	1.1E ± 06	1.1E ± 06		Heterogeneous nuclear ribonucleoproteins A2/B1
**MSN**	1.6E ± 05	2.2E ± 05	1.5E ± 05	1.8E ± 05	*	2.1E ± 05	2.3E ± 05	2.1E ± 05	2.2E ± 05		Moesin
**PYGB**	2.2E ± 04	3.2E ± 04	2.0E ± 04	2.6E ± 04	*	3.0E ± 04	3.8E ± 04	3.1E ± 04	3.0E ± 04		Glycogen phosphorylase, brain form
**RPS2**	2.4E ± 05	3.1E ± 05	2.6E ± 05	2.7E ± 05		3.2E ± 05	3.6E ± 05	3.2E ± 05	2.7E ± 05	*	40S ribosomal protein S2
**CAP1**	2.2E ± 05	3.2E ± 05	2.0E ± 05	2.5E ± 05	*	3.2E ± 05	3.7E ± 05	3.0E ± 05	2.5E ± 05	*	Adenylyl cyclase-associated protein 1
**HSPA2**	4.3E ± 05	4.9E ± 05	3.9E ± 05	4.3E ± 05	*	5.0E ± 05	5.5E ± 05	4.7E ± 05	4.5E ± 05		Heat shock-related 70 kDa protein 2
**KIF5B**	6.6E ± 04	7.6E ± 04	5.9E ± 04	7.0E ± 04	*	8.3E ± 04	9.1E ± 04	8.0E ± 04	8.8E ± 04		Kinesin-1 heavy chain
**PPME1**	4.5E ± 04	6.2E ± 04	3.9E ± 04	5.0E ± 04	*	5.6E ± 04	6.6E ± 04	5.6E ± 04	5.8E ± 04		Protein phosphatase methylesterase 1

**TUBB4**	4.4E ± 06	5.8E ± 06	4.0E ± 06	4.7E ± 06	*	5.6E ± 06	6.9E ± 06	5.2E ± 06	5.7E ± 06		Tubulin beta-4B chain
**ALDH16A1**	8.7E ± 03	1.2E ± 04	9.6E ± 03	1.0E ± 04		1.1E ± 04	1.4E ± 04	1.3E ± 04	1.1E ± 04		Aldehyde dehydrogenase family 16 member A1
**C14orf166**	3.4E ± 04	4.4E ± 04	3.4E ± 04	3.9E ± 04		4.2E ± 04	4.2E ± 04	4.2E ± 04	3.2E ± 04	*	RNA transcription, translation and transport factor protein
**CAND1**	2.5E ± 04	3.3E ± 04	2.9E ± 04	2.7E ± 04		3.2E ± 04	4.3E ± 04	3.5E ± 04	3.4E ± 04		Cullin-associated NEDD8-dissociated protein 1
**CLTC**	4.9E ± 05	5.4E ± 05	4.5E ± 05	4.8E ± 05		5.7E ± 05	6.2E ± 05	5.5E ± 05	5.4E ± 05		Clathrin heavy chain 1
**DES**	1.3E ± 05	1.5E ± 05	1.2E ± 05	1.3E ± 05		1.7E ± 05	1.8E ± 05	1.5E ± 05	1.4E ± 05		Desmin
**EIF2A**	5.1E ± 04	5.9E ± 04	4.7E ± 04	5.2E ± 04		7.0E ± 04	6.7E ± 04	5.9E ± 04	5.6E ± 04		Eukaryotic translation initiation factor 2A
**EIF3E**	7.8E ± 04	8.8E ± 04	8.2E ± 04	7.5E ± 04		9.8E ± 04	1.1E ± 05	8.7E ± 04	7.4E ± 04	*	Eukaryotic translation initiation factor 3 subunit E
**EIF3H**	6.1E ± 04	6.9E ± 04	6.2E ± 04	6.3E ± 04		7.1E ± 04	8.0E ± 04	6.9E ± 04	5.9E ± 04	*	Eukaryotic translation initiation factor 3 subunit H
**EIF3K**	5.0E ± 04	6.4E ± 04	5.0E ± 04	5.3E ± 04		7.0E ± 04	7.3E ± 04	6.3E ± 04	5.9E ± 04		Eukaryotic translation initiation factor 3 subunit K
**FASN**	4.9E ± 05	6.1E ± 05	5.2E ± 05	5.5E ± 05		6.7E ± 05	7.1E ± 05	6.8E ± 05	5.7E ± 05	*	Fatty acid synthase
**HNRNPA1L2**	9.1E ± 05	1.1E ± 06	8.5E ± 05	1.1E ± 06		9.4E ± 05	1.1E ± 06	1.1E ± 06	9.8E ± 05		Heterogeneous nuclear ribonucleoprotein A1-like 2
**IPO4**	9.4E ± 04	1.1E ± 05	8.3E ± 04	9.2E ± 04		1.2E ± 05	1.4E ± 05	1.1E ± 05	1.2E ± 05		Importin-4
**NAP1L1**	1.1E ± 05	1.4E ± 05	1.1E ± 05	1.2E ± 05		1.4E ± 05	1.7E ± 05	1.3E ± 05	1.3E ± 05		Nucleosome assembly protein 1-like 1
**PGD**	1.3E ± 05	1.8E ± 05	1.3E ± 05	1.3E ± 05		1.7E ± 05	2.2E ± 05	1.7E ± 05	1.7E ± 05		6-phosphogluconate dehydrogenase, decarboxylating
**PSMD13**	2.4E ± 05	2.7E ± 05	2.2E ± 05	2.4E ± 05		3.0E ± 05	3.3E ± 05	2.9E ± 05	2.8E ± 05		Proteasome 26S subunit, non-ATPase 13
**PSME1**	1.5E ± 05	1.9E ± 05	1.5E ± 05	1.7E ± 05		2.0E ± 05	2.3E ± 05	2.0E ± 05	1.7E ± 05	*	Proteasome activator complex subunit 1
**RPL15**	4.5E ± 04	6.6E ± 04	4.3E ± 04	5.0E ± 04		7.0E ± 04	8.8E ± 04	8.7E ± 04	5.2E ± 04	*	Ribosomal protein L15
**SMC4**	1.6E ± 04	2.1E ± 04	1.8E ± 04	1.6E ± 04		2.1E ± 04	2.9E ± 04	2.3E ± 04	1.9E ± 04		Structural maintenance of chromosomes protein 4
**SRP68**	4.9E ± 04	6.6E ± 04	5.3E ± 04	5.8E ± 04		6.8E ± 04	6.9E ± 04	6.2E ± 04	6.2E ± 04		Signal recognition particle subunit SRP68
**XRCC6**	5.7E ± 05	6.5E ± 05	6.2E ± 05	5.9E ± 05		6.8E ± 05	6.8E ± 05	6.4E ± 05	5.9E ± 05		X-ray repair cross-complementing protein 6
**ATAD3**	7.8E ± 04	6.8E ± 04	8.3E ± 04	7.3E ± 04	*	8.4E ± 04	7.2E ± 04	7.2E ± 04	6.3E ± 04		ATPase family AAA domain-containing protein 3
**CS**	2.9E ± 05	2.6E ± 05	3.0E ± 05	2.7E ± 05		2.9E ± 05	2.7E ± 05	2.7E ± 05	2.3E ± 05	*	Citrate synthase, mitochondrial
**PHB2**	5.2E ± 05	4.8E ± 05	5.4E ± 05	5.0E ± 05		5.6E ± 05	4.7E ± 05	5.4E ± 05	4.7E ± 05	*	Prohibitin-2
**SURF4**	2.0E ± 05	1.6E ± 05	1.8E ± 05	1.8E ± 05		2.4E ± 05	1.8E ± 05	2.0E ± 05	1.5E ± 05	*	Surfeit locus protein 4
**YLPM1**	8.9E ± 04	7.7E ± 04	9.0E ± 04	8.3E ± 04		8.8E ± 04	7.8E ± 04	8.4E ± 04	7.1E ± 04	*	YLP motif-containing protein 1
**ATL3**	3.3E ± 04	3.2E ± 04	4.6E ± 04	3.5E ± 04	*	4.4E ± 04	4.1E ± 04	4.6E ± 04	3.8E ± 04	*	Atlastin-3

**EIF3A**	1.3E ± 05	1.4E ± 05	1.5E ± 05	1.2E ± 05	*	1.6E ± 05	1.8E ± 05	1.5E ± 05	1.2E ± 05	*	Eukaryotic translation initiation factor 3 subunit A
**HSPD1**	2.1E ± 06	2.0E ± 06	2.4E ± 06	2.1E ± 06	*	2.4E ± 06	2.0E ± 06	2.2E ± 06	1.8E ± 06	*	60 kDa heat shock protein, mitochondrial
**KRAS**	1.7E ± 04	1.6E ± 04	2.1E ± 04	1.6E ± 04	*	1.4E ± 04	1.6E ± 04	1.7E ± 04	1.2E ± 04	*	GTPase KRas
**NCL**	1.4E ± 06	1.4E ± 06	1.6E ± 06	1.4E ± 06	*	1.5E ± 06	1.5E ± 06	1.5E ± 06	1.3E ± 06	*	Nucleolin
**PON2**	7.4E ± 04	6.8E ± 04	8.4E ± 04	6.7E ± 04	*	7.8E ± 04	6.8E ± 04	7.5E ± 04	6.6E ± 04		Serum paraoxonase/arylesterase 2
**PPA2**	7.0E ± 04	6.9E ± 04	8.0E ± 04	7.2E ± 04	*	7.1E ± 04	6.3E ± 04	6.8E ± 04	5.8E ± 04	*	Inorganic pyrophosphatase 2, mitochondrial
**RAB10**	7.8E ± 04	7.6E ± 04	9.1E ± 04	7.7E ± 04	*	9.0E ± 04	8.7E ± 04	1.0E ± 05	8.5E ± 04	*	Ras-related protein Rab-10
**TXNDC5**	1.7E ± 05	1.6E ± 05	1.9E ± 05	1.6E ± 05	*	1.8E ± 05	1.6E ± 05	1.7E ± 05	1.5E ± 05	*	Thioredoxin domain-containing protein 5
**ALDH18A1**	6.5E ± 04	6.9E ± 04	7.6E ± 04	6.9E ± 04		7.4E ± 04	6.7E ± 04	7.5E ± 04	6.2E ± 04	*	Delta-1-pyrroline-5-carboxylate synthase
**DDX3X**	7.2E ± 04	8.3E ± 04	8.4E ± 04	7.5E ± 04		8.7E ± 04	9.7E ± 04	8.2E ± 04	7.2E ± 04		ATP-dependent RNA helicase DDX3X
**NDUFS8**	1.2E ± 05	1.2E ± 05	1.4E ± 05	1.3E ± 05		1.3E ± 05	1.1E ± 05	1.3E ± 05	9.7E ± 04	*	NADH dehydrogenase [ubiquinone] iron-sulfur protein 8, mitochondrial
**SKIV2L2**	5.6E ± 04	5.8E ± 04	6.6E ± 04	5.9E ± 04		7.0E ± 04	6.7E ± 04	6.8E ± 04	5.7E ± 04	*	Superkiller viralicidic activity 2-like 2
**ATL2**	2.8E ± 05	3.1E ± 05	2.3E ± 05	2.6E ± 05		3.0E ± 05	3.9E ± 05	2.9E ± 05	3.2E ± 05		Atlastin-2
**TOMM5**	1.9E ± 05	2.0E ± 05	1.5E ± 05	1.7E ± 05		2.0E ± 05	2.4E ± 05	1.6E ± 05	2.0E ± 05	*	Mitochondrial import receptor subunit TOM5 homolog
**USP9X**	2.1E ± 04	2.4E ± 04	1.7E ± 04	1.9E ± 04		2.8E ± 04	2.9E ± 04	2.7E ± 04	2.3E ± 04		Probable ubiquitin carboxyl-terminal hydrolase FAF-X
**ACSS3**	1.5E ± 05	1.4E ± 05	9.5E ± 04	1.2E ± 05		1.3E ± 05	1.3E ± 05	1.4E ± 05	1.1E ± 05		Acyl-CoA synthetase short-chain family member 3, mitochondrial
**NPM1**	1.5E ± 06	1.4E ± 06	1.6E ± 06	1.3E ± 06	*	1.5E ± 06	1.3E ± 06	1.4E ± 06	9.3E ± 05	*	Nucleophosmin
**BAX**	5.5E ± 04	6.1E ± 04	4.7E ± 04	4.5E ± 04		5.9E ± 04	6.6E ± 04	5.6E ± 04	4.6E ± 04	*	Apoptosis regulator BAX
**CAB39**	8.4E ± 04	7.9E ± 04	7.9E ± 04	6.5E ± 04		7.4E ± 04	1.0E ± 05	7.5E ± 04	6.4E ± 04		Calcium-binding protein 39
**ANXA2**	9.2E ± 05	1.1E ± 06	1.3E ± 06	1.0E ± 06	*	1.0E ± 06	1.2E ± 06	1.1E ± 06	1.0E ± 06		Annexin A2
**SPTAN1**	3.0E ± 04	4.1E ± 04	4.0E ± 04	3.6E ± 04		4.3E ± 04	4.4E ± 04	4.5E ± 04	3.1E ± 04	*	Spectrin alpha chain, non-erythrocytic 1
**EIF3L**	2.6E ± 04	2.5E ± 04	2.8E ± 04	2.3E ± 04	*	2.8E ± 04	2.6E ± 04	2.7E ± 04	2.0E ± 04	*	Eukaryotic translation initiation factor 3 subunit L
**HTATIP2**	3.3E ± 04	3.6E ± 04	3.7E ± 04	3.1E ± 04	*	4.2E ± 04	3.9E ± 04	4.2E ± 04	3.9E ± 04		Oxidoreductase HTATIP2
**LRPPRC**	8.6E ± 05	8.2E ± 05	9.5E ± 05	8.5E ± 05	*	9.4E ± 05	8.3E ± 05	8.8E ± 05	7.0E ± 05	*	Leucine-rich PPR motif-containing protein, mitochondrial
**POTEI**	2.7E ± 04	3.2E ± 04	2.1E ± 04	3.1E ± 04	*	2.5E ± 04	3.7E ± 04	3.3E ± 04	3.0E ± 04		POTE ankyrin domain family member I

**SNRNP200**	6.8E ± 04	7.0E ± 04	6.0E ± 04	7.0E ± 04	*	7.3E ± 04	7.6E ± 04	7.2E ± 04	5.9E ± 04	*	U5 small nuclear ribonucleoprotein 200 kDa helicase
**ATAD3A**	1.9E ± 04	2.3E ± 04	1.6E ± 04	1.7E ± 04		2.0E ± 04	2.5E ± 04	2.1E ± 04	2.1E ± 04		ATPase family AAA domain-containing protein 3A
**EFTUD2**	1.1E ± 05	1.2E ± 05	1.0E ± 05	1.1E ± 05		1.1E ± 05	1.2E ± 05	1.1E ± 05	1.1E ± 05		116 kDa U5 small nuclear ribonucleoprotein component
**EZR**	4.3E ± 04	4.9E ± 04	4.1E ± 04	4.1E ± 04		5.2E ± 04	5.9E ± 04	4.8E ± 04	3.8E ± 04	*	Ezrin
**MARS**	1.9E ± 04	2.1E ± 04	1.6E ± 04	1.6E ± 04		2.3E ± 04	2.4E ± 04	2.0E ± 04	2.0E ± 04		MAGUK p55 subfamily member 5
**SSB**	2.3E ± 05	2.6E ± 05	2.4E ± 05	2.2E ± 05		2.7E ± 05	2.9E ± 05	2.5E ± 05	2.2E ± 05	*	Lupus La protein
**TRAP1**	2.3E ± 05	2.1E ± 05	2.5E ± 05	2.2E ± 05		2.5E ± 05	2.1E ± 05	2.3E ± 05	1.9E ± 05	*	Heat shock protein 75 kDa, mitochondrial
**CLINT1**	1.6E ± 05	1.7E ± 05	1.7E ± 05	1.5E ± 05		1.9E ± 05	1.8E ± 05	1.6E ± 05	1.2E ± 05	*	Clathrin interactor 1
**EHD2**	5.2E ± 04	5.6E ± 04	4.9E ± 04	5.0E ± 04		6.9E ± 04	7.3E ± 04	6.6E ± 04	5.8E ± 04		EH domain-containing protein 2
**KRT10**	6.9E ± 04	7.0E ± 04	5.8E ± 04	6.0E ± 04		1.1E ± 05	6.1E ± 04	6.4E ± 04	7.0E ± 04		Keratin. type I cytoskeletal 10
**MAPRE1**	1.6E ± 05	1.6E ± 05	1.7E ± 05	1.6E ± 05		1.7E ± 05	1.7E ± 05	1.7E ± 05	1.3E ± 05	*	Microtubule-associated protein RP/EB family member 1
**MYOF**	2.8E ± 05	2.7E ± 05	2.9E ± 05	2.8E ± 05		3.6E ± 05	3.2E ± 05	3.5E ± 05	2.8E ± 05	*	Myoferlin
**NUP205**	2.8E ± 04	3.2E ± 04	3.0E ± 04	2.9E ± 04		3.9E ± 04	4.2E ± 04	4.0E ± 04	3.2E ± 04	*	Nuclear pore complex protein Nup205
**PRKDC**	2.8E ± 04	2.5E ± 04	2.5E ± 04	2.5E ± 04		2.4E ± 04	2.4E ± 04	2.5E ± 04	1.9E ± 04	*	DNA-dependent protein kinase catalytic subunit
**RAP1A**	2.4E ± 05	2.4E ± 05	2.6E ± 05	2.6E ± 05		3.1E ± 05	2.8E ± 05	3.2E ± 05	2.7E ± 05	*	Ras-related protein Rap-1A
**POTEE**	1.6E ± 04	1.6E ± 04	1.7E ± 04	1.7E ± 04		1.7E ± 04	1.6E ± 04	1.6E ± 04	1.2E ± 04	*	POTE ankyrin domain family member E
**SF3B1**	3.6E ± 05	3.7E ± 05	3.4E ± 05	3.7E ± 05		3.9E ± 05	4.0E ± 05	3.7E ± 05	3.3E ± 05		Splicing factor 3B subunit 1
**CNP**	3.6E ± 04	3.9E ± 04	3.7E ± 04	3.4E ± 04		4.2E ± 04	3.6E ± 04	3.6E ± 04	3.9E ± 04		2'.3'-cyclic-nucleotide 3'-phosphodiesterase
**CCT7**	1.0E ± 06	1.0E ± 06	9.3E ± 05	1.0E ± 06		1.1E ± 06	1.1E ± 06	1.1E ± 06	1.1E ± 06		T-complex protein 1 subunit eta
**DDX18**	6.7E ± 04	6.7E ± 04	6.1E ± 04	6.7E ± 04		7.2E ± 04	8.2E ± 04	7.4E ± 04	6.5E ± 04		ATP-dependent RNA helicase DDX18
**HIST1H2B**	2.3E ± 07	2.4E ± 07	1.3E ± 07	1.4E ± 07		2.9E ± 06	4.9E ± 06	1.7E ± 07	1.6E ± 07		Histone H2B type 1-B
**IKBA**	1.4E ± 04	1.5E ± 04	1.4E ± 04	1.3E ± 04		1.1E ± 04	1.2E ± 04	1.3E ± 04	1.2E ± 04		NF-kappa-B inhibitor alpha
**KDM1A**	4.2E ± 04	4.6E ± 04	4.3E ± 04	4.2E ± 04		5.1E ± 04	5.3E ± 04	4.8E ± 04	4.6E ± 04		Lysine-specific histone demethylase 1A
**KRT18**	1.0E ± 06	1.0E ± 06	1.0E ± 06	9.3E ± 05		7.5E ± 05	7.9E ± 05	1.1E ± 06	8.4E ± 05		Keratin. type I cytoskeletal 18
**MYO1C**	4.2E ± 04	4.1E ± 04	3.3E ± 04	3.1E ± 04		5.0E ± 04	5.8E ± 04	5.9E ± 04	5.3E ± 04		Unconventional myosin-Ic
**PLEC**	2.2E ± 05	2.3E ± 05	1.7E ± 05	2.0E ± 05		2.4E ± 05	2.3E ± 05	2.5E ± 05	2.2E ± 05		Plectin
**VIM**	6.2E ± 05	6.7E ± 05	3.4E ± 05	4.3E ± 05		9.0E ± 04	7.9E ± 04	4.9E ± 05	4.7E ± 05		Vimentin
